# Predicting Gram-negative bloodstream infection in elderly patients after isolation of GNB from non-blood specimens: a machine learning-based tool

**DOI:** 10.3389/fmed.2026.1819369

**Published:** 2026-06-16

**Authors:** Xinran Lin, Daoming Zhang, Ping Jiang, Yongping Yao, Yu Lv

**Affiliations:** 1 School of Medicine, University of Electronic Science and Technology of China, Chengdu, Sichuan, China; 2 Sichuan Nursing Vocational College, Chengdu, Sichuan, China; 3 Public Health Department, Sichuan Academy of Medical Sciences, Sichuan People’s Hospital, School of Medicine, University of Electronic Science and Technology of China, Chengdu, Sichuan, China

**Keywords:** bloodstream infection, elderly, Gram-negative bacteria, machine learning, predictive model, XGBoost

## Abstract

**Background:**

This study aimed to develop a machine learning-based model for the early identification of elderly inpatients at high risk of Gram-negative bloodstream infection immediately following the first detection of Gram-negative bacteria in non-blood specimens (e.g., urine, sputum, wound secretions), during the 48–72 h window before blood culture results become available.

**Methods:**

A retrospective cohort study was conducted across three centers of Sichuan Provincial People’s Hospital (Qingyang, Caotang, and Chengdong Campuses). It enrolled 9,646 elderly inpatients with Gram-negative bacteria positivity in any specimen during their hospitalization between January 2017 and December 2022. Predictor variables were screened using LASSO regression and Boruta algorithm, and their clinical significance was validated via the Delphi expert consultation method. Six machine learning models—random forest, XGBoost, logistic regression, artificial neural network, k-nearest neighbors, and decision tree—were developed. The discriminatory performance of the models was evaluated using a comprehensive set of metrics, including the area under the receiver operating characteristic curve along with other key indicators such as accuracy and recall.

**Results:**

The XGBoost model demonstrated the optimal performance among the developed models, achieving a test set AUC of 0.816 (95% CI: 0.766–0.861), accuracy of 0.733, recall of 0.760, while logistic regression offered a simpler alternative with acceptable accuracy. Seven predictive variables were identified: maximum procalcitonin level, max neutrophil percentage, maximum C-reactive protein level, minimum white blood cell count, venous catheter, age, and length of hospital stay. SHAP variable importance analysis identified maximum procalcitonin level, length of hospital stays, and max_neutrophil_percentage as the top three most important predictors. External validation in an independent MIMIC-IV database confirmed acceptable generalizability.

**Conclusion:**

The predictive model developed for Gram-negative bloodstream infections in elderly patients demonstrates promising predictive ability. It overcomes the temporal limitations of traditional blood culture, enabling the identification of high-risk elderly patients at the earliest stage of infection. This approach provides clinicians with a valuable intervention window and represents a novel pathway toward precision infection control.

## 1 Introduction

Bloodstream infections (BSI) represent a critical clinical condition with substantial disease burden. Globally, the incidence and mortality rates of BSI have remained persistently high, posing a significant public health challenge. In China, a nationwide multicenter study reported that the incidence of hospital-acquired BSI ranges from 1.3 to 6.5%, with a crude mortality rate as high as 30 to 40% (1). Annually, BSI is associated with approximately 80,000 and 150,000 deaths in North America and Europe, respectively, prolongs the length of hospital stay by 8 to 14 days on average, and increases healthcare costs by 2- to 3-fold (2). China, with the world’s largest aging population, is confronting unprecedented challenges posed by population aging (3, 4). Against this backdrop, elderly hospitalized patients often present with multiple chronic comorbidities, age-related immune dysfunction, high colonization rates of multidrug-resistant (MDR) organisms, and polypharmacy—complex clinical conditions that significantly increase susceptibility to infection and adverse outcomes. Consequently, they constitute a high-risk population for BSI. Moreover, the clinical manifestations of BSI in the elderly are frequently atypical and can be masked by symptoms of underlying chronic diseases, often leading to diagnostic delays and increased disease burden (5–7). Therefore, early identification and intervention for BSI in elderly patients are crucial for improving clinical outcomes and controlling healthcare costs.

Gram-negative bacteria (GNB) are the predominant pathogens causing BSI, and this predominance is particularly pronounced in the elderly population (8, 9). According to the 2024 CHINET (China Antimicrobial Resistance Surveillance Network) data, GNB account for a substantial proportion (65.7–73.0%) of clinically isolated bacterial strains in China. The detection rate, case fatality rate, and drug resistance rate of GNB are significantly higher than those of Gram-positive bacteria (GPB) (10, 11). The big three Gram-negative pathogens (*Pseudomonas aeruginosa*, *Acinetobacter baumannii*, and *Klebsiella pneumoniae*) pose a particular concern. These pathogens exhibit both inherent and readily acquired resistance mechanisms and frequently present as MDR strains, representing the core challenge of antimicrobial resistance (12). Currently, Gram-negative bacterial bloodstream infections (GNB-BSI) in elderly patients pose a severe public health challenge in China.

The effective management of GNB-BSI is highly dependent on the early initiation of appropriate antimicrobial therapy, since any delay significantly increases the risk of mortality (13, 14). Current clinical decision-making heavily relies on blood culture results, which typically involve a 48–72 h turnaround time (TAT) before confirming the pathogen and obtaining antimicrobial susceptibility data. This delay can render empirical antimicrobial therapy suboptimal and may lead to missed critical intervention windows (15, 16). Consequently, there is an urgent need for a tool that can rapidly identify elderly inpatients at high risk for GNB-BSI using readily available clinical indicators before blood culture results are available, thereby overcoming the therapeutic delays associated with the current culture-dependent approach.

Traditional predictive models, which are predominantly linear, struggle to accurately capture the unique pathogenetic and clinical characteristics of GNB-BSI (17), and are even less effective in modeling the complex and often atypical clinical presentations seen in elderly patients. Machine learning algorithms, by contrast, excel at identifying complex, high-dimensional, and nonlinear relationships, thus presenting a novel approach to this challenge. Therefore, this study aimed to develop and validate an early risk prediction model for GNB-BSI in elderly inpatients (age ≥ 65 years) by applying machine learning algorithms to large-scale, multicenter, retrospective clinical data. The model was designed to systematically integrate multidimensional features, including demographics, comorbidities, therapeutic interventions, and laboratory indicators, to develop a high-performance predictive tool. This tool is intended to identify high-risk patients early in the course of infection, thereby securing a critical intervention window for clinicians and providing new decision support for precision infection control.

## 2 Materials and methods

### 2.1 Subjects

**Figure 1:**
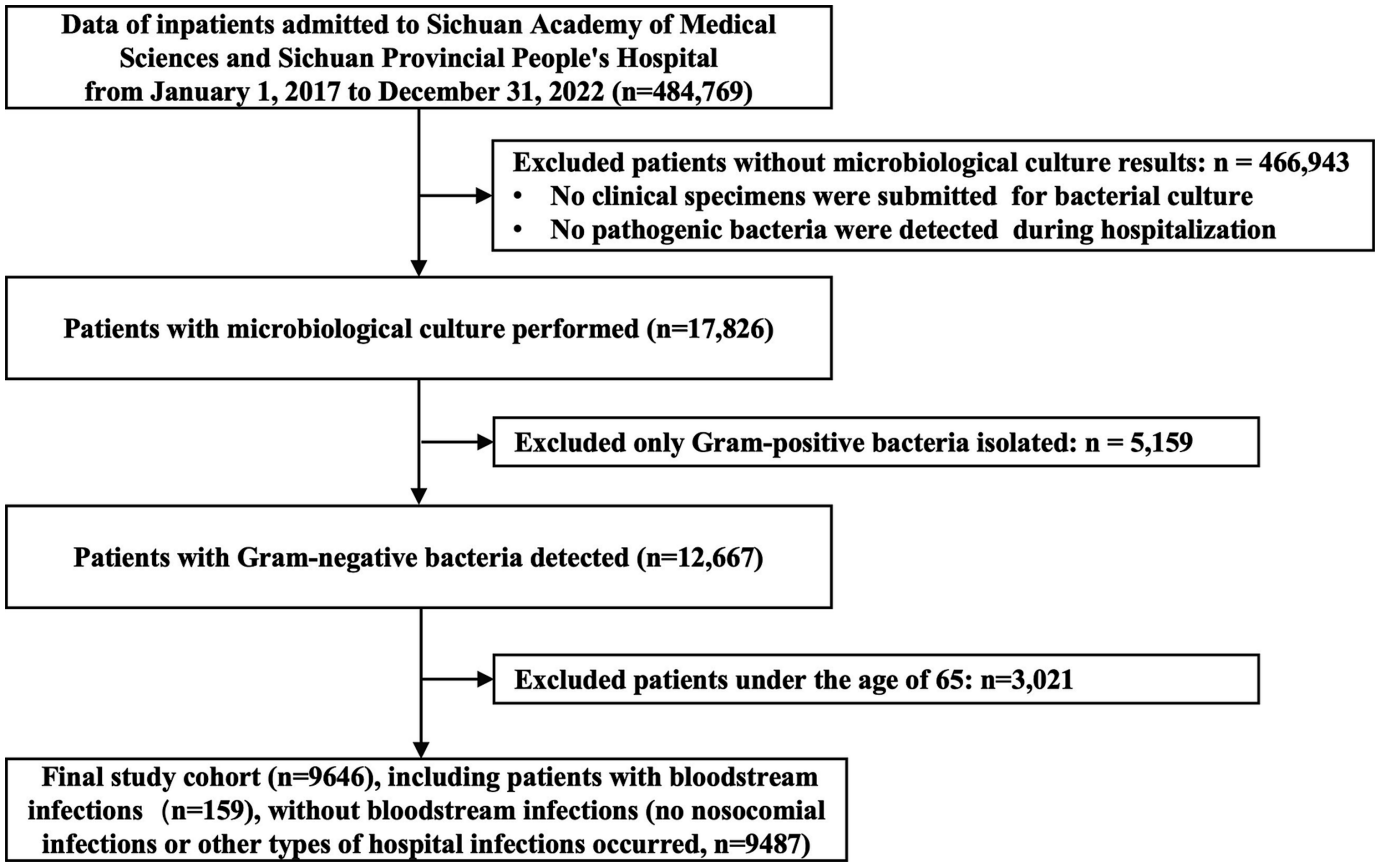
Flowchart of study patient selection. Summarizes patient inclusion/exclusion. From 484,769 inpatients, those without microbiological culture results were excluded. After applying GNB detection and age ≥65 years, 9,646 elderly patients were included, of whom 159 developed GNB-BSI and 9,487 did not.

**Figure 2:**
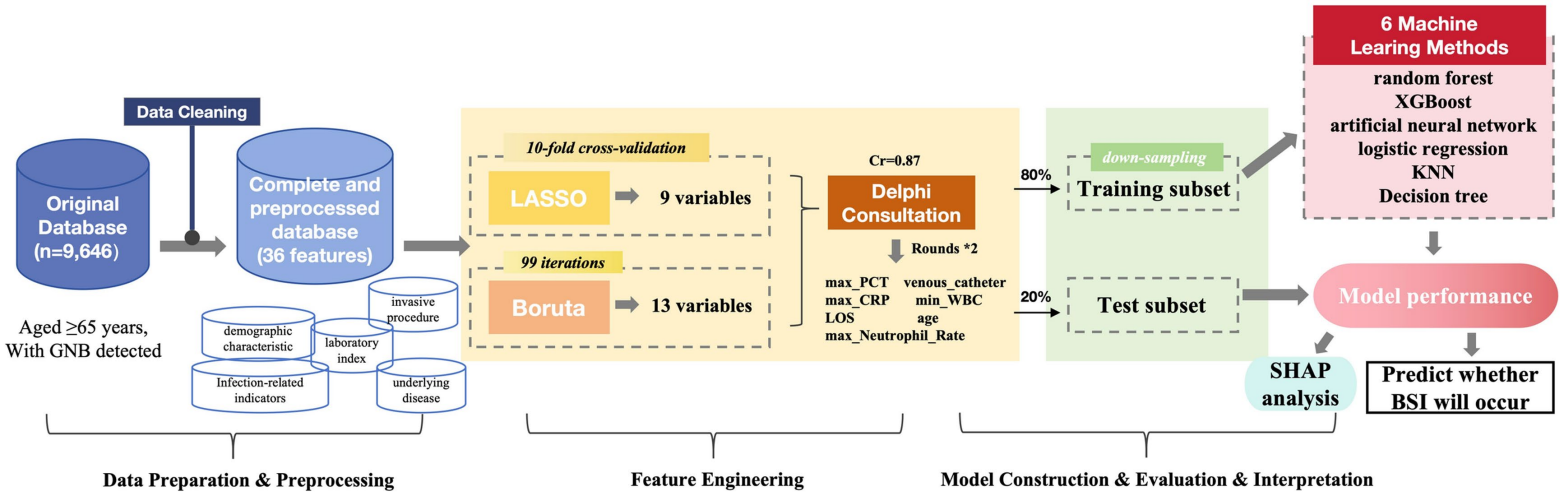
Overall workflow for developing and validating the prediction model for Gram-negative bacterial bloodstream infection. The pipeline includes: data collection (2017–2022, three campuses), feature selection (LASSO, Boruta, Delphi consensus), six machine learning models, internal validation (repeated random undersampling and SMOTE), external validation (MIMIC-IV database), and SHAP interpretation. The final outputs are a risk prediction tool with a recommended threshold and an actionable clinical pathway.

This retrospective cohort study collected data from 484,769 inpatients admitted to the Sichuan Medical Academy and Sichuan Provincial People’s Hospital Multicenter Medical Alliance between January 1, 2017, and December 31, 2022. This hospital is a modern comprehensive tertiary care center that integrates medical care, teaching, research, and preventive health services. It operates over 4,500 beds across multiple campuses, including the Qingyang (main), Caotang (Sichuan Provincial Geriatric Medical Center), and Chengdong (East) Campuses. Its critical care system comprises more than 200 intensive care unit (ICU) beds, including general, specialty, and emergency ICUs. After excluding 466,943 patients with no microbiological culture results (either no specimens submitted during hospitalization or specimens submitted but no pathogens detected), 17,826 cases remained. Not all inpatients underwent microbiological testing; cultures were ordered by attending physicians based on clinical suspicion of infection (e.g., fever, leukocytosis, focal signs). Further excluding 5,159 patients who isolated only Gram-positive bacteria (GPB), 12,667 patients were identified who had GNB detected for the first time in any clinical specimen (e.g., sputum, urine, wound secretions) during hospitalization. Ultimately, 9,646 elderly patients (age≥65 years) were included in the final study cohort. A flowchart detailing the patient selection process is presented in Figure 1. Based on infection status, patients were categorized into the GNB-BSI group (n = 159) and the non-GNB-BSI group (n = 9,487). The overall workflow for developing and validating the prediction model is summarized in Figure 2. The study protocol was reviewed and approved by the Ethics Committee of Sichuan Medical Academy and Sichuan Provincial People’s Hospital (Supplementary File S1).

### 2.2 Infection diagnosis

#### 2.2.1 Inclusion criteria for GNB-BSI

(1) Detection of pathogenic GNB in blood cultures; (2) Meeting criteria for vascular-associated infections, sepsis, or transfusion-associated infections as outlined in the Ministry of Health’s “Diagnostic Criteria for Hospital-Acquired Infections (Trial)” (18), with specific operational definitions detailed in Supplementary Table S1; (3) Prospective continuous monitoring of all inpatients using the BSI Prospective Whistleblower System(software copyright registration provided in Supplementary File S3) developed by the Sichuan Provincial Hospital Infection Quality Control Center. The system’s intelligent recognition program automatically screens for suspected BSI cases meeting any of the following criteria: ① Fever (>38 °C), ② Hypotension (systolic pressure <90 mmHg and/or diastolic pressure <60 mmHg), ③ Oliguria (<400 mL/day), ④ Detection of microorganisms in ≥1 blood culture, ⑤ Cases entered prospectively into the hospital infection electronic system by physicians. This system holds a computer software copyright registration from the National Copyright Administration (Registration No. 9513151); (4) Cases flagged by the system undergo independent assessment and confirmation by two infectious disease physicians with the title of associate chief physician or above.

#### 2.2.2 Exclusion criteria for GNB-BSI

(1) Single positive blood culture without supporting clinical symptoms; (2) Laboratory records indicating potential specimen contamination; (3) Clinical data confirming community-acquired infection.

### 2.3 Data collection

This study encompasses clinical data from the hospital information system, ICU system, laboratory information system, and hospital infection surveillance system. Key research variables include the following aspects: patient demographics (age, gender), infection-related indicators (community-infection status at admission, presence of MDR organism infection, bacteria_class [Enterobacteriaceae, *Pseudomonas aeruginosa*, *Acinetobacter baumannii*, others]), ICU admission status, length of stay (LOS, which is calculated as the time from hospital admission to the date of first GNB isolation from non-blood specimens), laboratory indicators (maximum white blood cell count[max_wbc], minimum white blood cell count[min_wbc], maximum platelet count[max_platelet], maximum neutrophil percentage[max_neutrophil_percentage], maximum C-reactive protein level[max_crp], maximum procalcitonin level[max_pct], maximum total protein level[max_total_protein], minimum albumin level[min_albumin]), invasive procedures (blood_transfusion, urinary_catheter, venous_catheter, mechanical_ventilation, tracheostomy, surgery), and underlying conditions (including hypertension, diabetes, copd, liver_failure, kidney_failure, heart_failure, and respiratory_failure). All variable data were collected from records on or before the day of the first detection of GNB in non-blood specimens (e.g., urine, sputum, wound secretions), the required maxima/minima are simply the highest or lowest values of each test recorded from admission to the time of GNB detection, readily retrievable from the electronic health record. This ensures that all predictors were available at the intended time of model application, which is immediately after non-blood GNB detection and before blood culture results become available. All data collection was performed by professionally trained researchers, with data quality verification conducted through random sampling to ensure the accuracy and reliability of the study data. Strict quality control measures were applied during data processing to address missing or anomalous data, safeguarding the scientific validity and credibility of the research findings.

### 2.4 Bacterial identification and antimicrobial susceptibility testing

Strains were identified using the VITEK 2 automated system (BioMérieux, France). Antimicrobial susceptibility testing was performed using both automated methods (with dedicated susceptibility cards) and the disk diffusion method (K-B method). Results were interpreted according to the 2023 Clinical and Laboratory Standards Institute (CLSI) standard (M100) (19). Interpretation of results for special agents (e.g., tigecycline, polymyxin) followed respective drug labels and domestic expert consensus guidelines (20, 21). Quality control was implemented throughout the testing process using reference strains including *Escherichia coli* ATCC 25922, *Escherichia coli* ATCC 35218, and *Pseudomonas aeruginosa* ATCC 27853. All quality control strains were purchased from the Clinical Laboratory Center of the Ministry of Health.

### 2.5 Statistical analysis

Data organization and statistical analysis were performed using R software (version 4.4.1). The dataset was randomly split into training and testing sets at an 8:2 ratio. The proportion of missing data for each candidate variable is presented in Supplementary Table S3. Missing values in continuous variables were imputed using median imputation via the impute function in the Hmisc package. Missing values in categorical variables were imputed using mode imputation. Multi-categorical variables (pathogen species) were treated as dummy variables.

Downsampling was employed to balance the positive-to-negative sample ratio in the training set. Random undersampling of the majority class was performed exclusively on the training set; the test set remained unchanged. All positive samples were retained, and an equal number of negative samples were randomly drawn from the training set, achieving a 1:1 ratio between the two classes. For each of the six machine learning models, the undersampling process was repeated five times with different random seeds. For each repetition, the model was trained on the balanced training set and evaluated on the unchanged test set. SMOTE (Synthetic Minority Oversampling Technique) was applied as an alternative balancing method using the “ovun.sample” function from the “ROSE” R package, with “method = ‘both’” and “*p* = 0.5,” to achieve a 1:1 balanced training set. SMOTE was applied exclusively to the training set; the test set remained unchanged. And the procedure was also repeated five times with different random seeds.

Quantitative data are presented as mean ± standard deviation (SD) or median (interquartile range). Comparisons were performed using t-tests or Mann–Whitney U tests (Wilcoxon signed-rank test). Relative frequencies were expressed as frequencies (percentages). Group comparisons used χ^2^ tests. All tests were two-tailed with a significance level of *α* = 0.05. During feature selection, the glmnet package performed Least Absolute Shrinkage and Selection Operator (LASSO) regression analysis, selecting the optimal *λ* value via 10-fold cross-validation. Concurrently, the Boruta package assessed variable importance, confirming key variables after 99 iterations. Six predictive models were constructed using the randomForest package (random forest), xgboost package (eXtreme Gradient Boosting [XGBoost]), glm function (logistic regression), nnet package (artificial neural network), caret package (k-nearest neighbors algorithm), and rpart package (decision tree). All machine learning models were trained with fixed hyperparameters to ensure reproducibility; the detailed parameter settings for each model are provided in Supplementary Table S7. Model evaluation involved plotting Receiver Operating Characteristic (ROC) curves using the pROC package, calculating Area Under the ROC Curve (AUC). To assess the stability of model performance and provide robust estimates of uncertainty, 95% confidence intervals for AUC were calculated using bootstrap resampling with 2000 iterations via the ci.auc function in the pROC package. The optimal probability threshold for classifying high-risk patients was determined using the Youden index (maximizing sensitivity + specificity – 1) based on the internal test set. This threshold can be adjusted upward or downward according to local clinical priorities. Additional performance metrics including accuracy, sensitivity, and specificity were also reported. The SHapley Additive exPlanations (SHAP) package was employed for variable contribution analysis to assess the direction and magnitude of each feature’s impact on prediction outcomes.

### 2.6 Clinical significance validation of predictive variables using the Delphi method

#### 2.6.1 Expert selection

To enhance the clinical applicability and acceptability of the predictive model, this study employed the Delphi expert consultation method to validate the clinical importance of variables, following an initial screening based on machine learning algorithms. Expert selection criteria were: (1) Employment at a provincial-level tertiary Class A hospital; (2) Possession of at least 5 years of clinical experience in infectious diseases, critical care medicine, or geriatrics; (3) Holding an associate senior or higher professional technical title. Ultimately, 13 experts were selected.

#### 2.6.2 Questionnaire development and consultation process

The two-round Delphi consultation questionnaires used in this study were specifically developed for this research (see Supplementary Files S4, S5). The expert consultation form employed a 9-point Likert scale. To ensure consistent interpretation, the scale was anchored with defined ranges: scores of 1–3 represented “not important” 4–6 “moderately important” and 7–9 “important” (1 = “Very Unimportant” 9 = “Very Important”). Consensus criteria were defined as ≥75% of experts scoring an item between 7 and 9, indicating consensus on its importance (22). The first round of expert consultation was conducted on September 6, 2025. The questionnaire (Supplementary File S4) initially included predictive variables selected via LASSO regression and Boruta algorithm, with experts rating their clinical importance. The second round of expert consultation was conducted on October 21, 2025. The questionnaire for the second round (Supplementary File S5) revised and clarified variable descriptions based on the first round’s expert ratings and qualitative modification suggestions. The statistical distribution of expert opinions was anonymously fed back to each expert for re-evaluation.

#### 2.6.3 Questionnaire analysis methods

Expert questionnaire results were analyzed using Excel 2019 and SPSS 29.0 software. Consultation quality indicators were assessed as follows: (1) Expert engagement: measured by valid questionnaire return rate. (2) Expert authority: calculated as the arithmetic mean of judgment basis (four dimensions: practical experience, theoretical analysis, etc.) and familiarity level (high, medium, low), as detailed in Table 1. An authority coefficient >0.7 indicates reliable consultation results (23). (3) Expert opinion coordination: Assessed using the coefficient of variation (CV) and Kendall’s coefficient of concordance (Kendall’s W), supplemented by χ^2^ testing. This study adopted CV ≤ 15% in the second consultation round as the criterion for consensus on variable significance (24, 25).

**Table 1:** Criteria for assessing expert authority.

Basis of judgment (Ca)				Level of familiarity (Cs)	
Degree	Assignment			Degree	Assignment
	Significant	Moderate	Weak	Very familiar	1.0
Practical experience	0.5	0.4	0.3	Relatively familiar	0.8
Theoretical analysis	0.3	0.2	0.1	Moderately familiar	0.6
Literature analysis	0.1	0.1	0.1	Slightly familiar	0.4
Intuitive feeling	0.1	0.1	0.1	Unfamiliar	0.2
Formula	Cr = (Ca + Cs) / 2				

### 2.7 External validation

To assess the generalizability of our model, we performed external validation using the MIMIC-IV database (version 2.2) (26). Access to the data was granted via the PhysioNet platform (27). The same inclusion criteria (age ≥ 65 years, first isolation of GNB from non-blood specimens) were applied. Because PCT was not available in MIMIC-IV, the external validation used the six remaining predictors: age, venous_catheter, LOS, min_wbc, max_crp, and max_neutrophil_percentage. Missing values in these continuous variables were imputed using the median; categorical variables had no missing data. The logistic regression model (trained on the internal development cohort, which is less sensitive to missing predictors) were directly applied to the MIMIC-IV cohort without any retraining or adjustment. Discrimination was evaluated using AUC, recall, accuracy, and specificity.

## 3 Results

During the study period, a total of 9,646 patients were enrolled, including 159 in the GNB-BSI group and 9,487 in the non-GNB-BSI group, with a GNB-BSI incidence rate of 1.68%. As shown in Table 2, patients in the GNB-BSI group were younger (Z = -2.905, *p* = 0.004) and had longer LOS (Z = 5.393, *p* = 0.001). They also exhibited higher rates of ICU admission (χ^2^ = 7.994, *p* = 0.005), blood_transfusion (χ^2^ = 71.198, *p* < 0.001), urinary_catheter (χ^2^ = 5.178, *p* = 0.023), and venous_catheter (χ^2^ = 62.217, *p* < 0.001) rates were significantly higher. Laboratory indicators showed that the GNB-BSI group had significantly higher max_wbc, max_crp, max_pct, and max_neutrophil_percentage compared to the non-GNB-BSI group (p < 0.001), while min_albumin (*p* = 0.003) and min_wbc (p < 0.001) were lower. The detection rate of MDR bacteria was higher in the GNB-BSI group (χ^2^ = 19.035, p < 0.001), and the bacterial species differed significantly between groups (χ^2^ = 8.403, *p* = 0.038).

**Table 2:** Baseline clinical characteristics of patients with GNB-BSI and non-GNB-BSI.

Variable	GNB_BSI_Group (*n* = 159)	Non_GNB_BSI_Group (*n* = 9,487)	Statistic	*p*-value
Gender-male (%)	98 (61.64%)	6,068 (63.96%)	0.273^*^	0.601
Age (years)	76.00 [69.00, 87.00]	79.00 [71.00, 88.00]	-2.905^†^	0.004
LOS (days)	26.00 [18.00, 35.00]	20.00 [12.00, 29.00]	5.393^†^	0.001
Community_infection (%)	24 (15.09%)	2,255 (23.77%)	6.050^*^	0.014
ICU_admission (%)	82 (51.57%)	3,810 (40.16%)	7.994^*^	0.005
Blood_transfusion (%)	101 (63.52%)	3,005 (31.67%)	71.198^*^	0.001
Urinary_catheter (%)	74 (46.54%)	3,549 (37.41%)	5.178^*^	0.023
Venous_catheter (%)	91 (57.23%)	2,689 (28.34%)	62.217^*^	0.001
Mechanical_ventilation (%)	70 (44.03%)	2,302 (24.26%)	31.872^*^	0.001
Tracheostomy (%)	26 (16.35%)	785 (8.27%)	12.222^*^	0.001
Hypertension (%)	73 (45.91%)	5,254 (55.38%)	5.294^*^	0.021
Diabetes (%)	38 (23.90%)	2,960 (31.20%)	3.558^*^	0.059
copd (%)	21 (13.21%)	1,966 (20.72%)	4.951^*^	0.026
liver_failure (%)	10 (6.29%)	187 (1.97%)	12.497^*^	0.001
renal_failure (%)	20 (12.58%)	789 (8.32%)	3.163^*^	0.075
heart_failure (%)	23 (14.47%)	1,230 (12.97%)	0.193^*^	0.661
respiratory_failure (%)	40 (25.16%)	2,155 (22.72%)	0.401^*^	0.527
Surgery (%)	127 (79.87%)	7,076 (74.59%)	2.041^*^	0.153
max_wbc(×10^9^/L)	11.75 [10.83, 16.73]	10.83 [7.76, 13.98]	5.894^†^	0.001
min_wbc(×10^9^/L)	4.87 [3.54, 5.71]	5.54 [4.46, 6.63]	−4.991^†^	0.001
max_neutrophil_percentage(%)	93.10 [89.10, 96.00]	85.80 [76.00, 92.30]	10.156^†^	0.001
max_crp(mg/L)	100.38 [73.23, 196.68]	55.89 [9.83, 96.38]	9.696^†^	0.001
max_pct(ng/mL)	7.52 [1.90, 33.49]	1.36 [0.41, 4.21]	10.536^†^	0.001
max_platelet(×10^9^/L)	215.00 [148.00, 300.00]	215.00 [154.00, 288.00]	0.588^†^	0.556
max_total_protein(g/L)	67.50 [62.40, 72.60]	67.20 [62.90, 71.90]	0.558^†^	0.577
min_albumin(g/L)	49.70 [37.60, 53.70]	53.70 [37.40, 56.70]	−2.980^†^	0.003
mdr_bacteria (%)	70 (44.03%)	2,656 (28.00%)	19.035^*^	0.001
Bacteria_class (%)			8.403^*^	0.038
Others	4 (2.52%)	575 (6.06%)		
bacteria_enterobacteriaceae	108 (67.92%)	5,889 (62.07%)		
bacteria_pseudomonas_aeruginosa	22 (13.84%)	1,864 (19.65%)		
bacteria_acinetobacter_baumannii	25 (15.72%)	1,159 (12.22%)		

†Wilcoxon rank-sum test. *Pearson’s chi-squared test.

**Figure 3:**
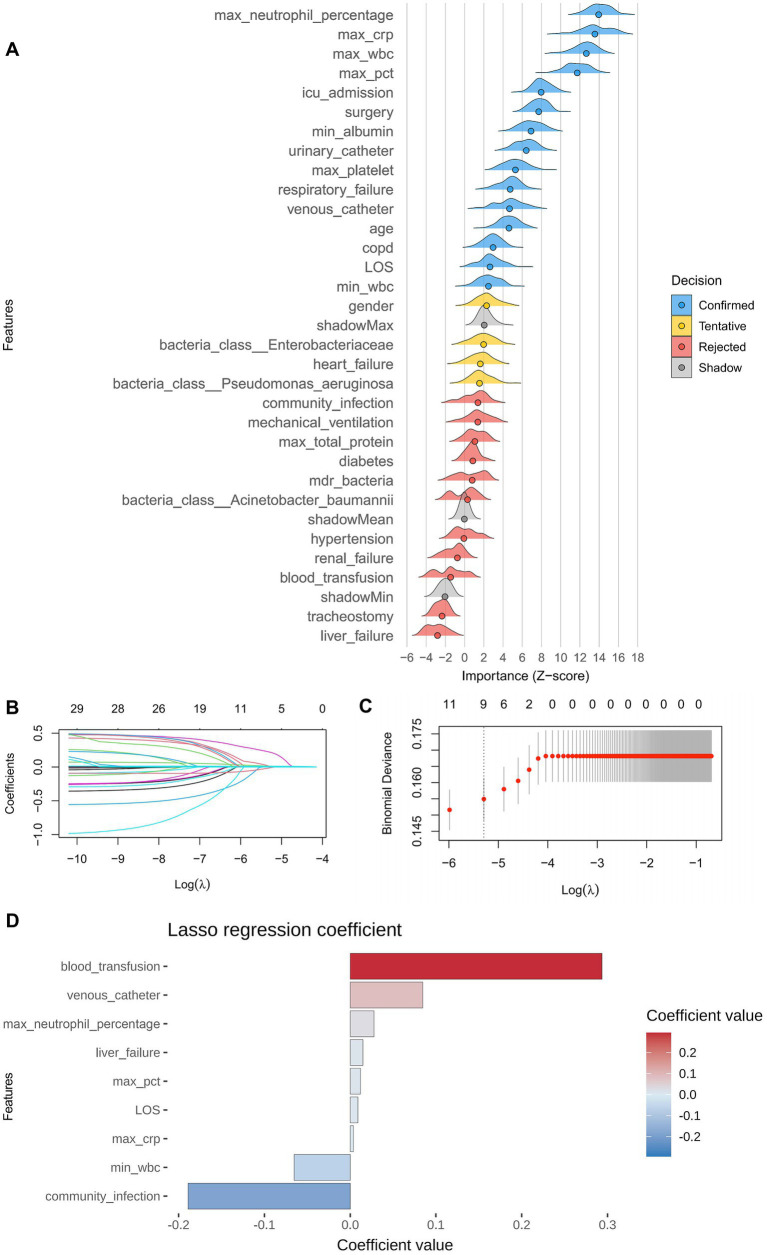
Machine learning feature selection process: **(A)** Boruta algorithm importance assessment; **(B)** LASSO regression coefficient trajectories as the regularization parameter log(*λ*) increases.; **(C)** 10-fold cross-validation error (partial likelihood deviance) versus log(λ); the optimal λ (0.00503) is marked by the left dashed line; **(D)** Final LASSO coefficients of the nine selected variables; positive values (e.g., blood_transfusion) increase GNB-BSI risk, negative values (e.g., min_wbc) decrease risk.

To screen core predictive variables, LASSO regression and the Boruta algorithm were employed for feature selection, with final variable inclusion determined through clinical expert assessment. As shown in Figure 3, the optimal *λ* value (λ = 0.00503) was determined through 10-fold cross-validation. LASSO regression identified 9 non-zero coefficient variables, among which blood_transfusion, venous_catheter and max_neutrophil_percentage showed significant positive correlations with GNB-BSI risk, while community_infection and min_wbc exhibited significant negative correlations. After 99 iterations, the Boruta algorithm confirmed 15 important variables. The importance ranking indicated max_neutrophil_percentage, max_crp, and max_wbc as the top three variables with the highest predictive value. Both methods jointly identified key inflammatory indicators such as max_pct and max_neutrophil_percentage, providing a crucial feature foundation for subsequent model construction. The corresponding coefficients (LASSO) and importance rankings (Boruta) for each candidate variable were provided to the experts in the Delphi questionnaires for reference (Supplementary Files S4, S5).

This study completed two rounds of Delphi expert consultations, distributing 13 questionnaires with a 100% valid response rate, indicating high expert engagement. The average authority coefficient was 0.79, demonstrating strong result reliability. Preliminary screening identified 18 candidate predictor variables by combining the results of LASSO regression (9 variables) and the Boruta algorithm (15 variables). Following two rounds of consultation, expert opinion coordination significantly improved (Kendall’s W: W = 0.372 in the first round, W = 0.446 in the second round, *p* < 0.001). In the second round, all variables meeting the high consensus criterion of CV ≤ 15% were evaluated. Ultimately, 7 variables—max_pct, max_neutrophil_percentage, max_crp, min_wbc, venous_catheter, age, and LOS—achieved high consensus (all CV < 15%) and were incorporated into the predictive model. Remaining variables (e.g., urinary_catheter, blood_transfusion, max_wbc, min_albumin) were excluded for failing to meet consensus criteria, as shown in Table 3.

**Table 3:** Final predictors selected via the Delphi method and their ratings.

Predictor	Mean	CV	Full score ratio
venous_catheter	7.769	0.141	30.77
max_pct	7.923	0.150	46.15
max_neutrophil_percentage	7.538	0.139	23.08
max_crp	7.615	0.147	23.08
LOS	7.538	0.139	23.08
age	7.615	0.137	15.38
min_wbc	7.462	0.141	23.08

To assess whether the Delphi-based exclusion of statistically identified variables affected model performance, we compared the final seven-variable XGBoost model with a statistics-only XGBoost model that included all 18 variables selected by LASSO and Boruta. As shown in Supplementary File S4 (Supplementary Figure S1), the AUC for the statistics-only model was 0.805 (95% CI: 0.725–0.757), compared to 0.816 (95% CI: 0.766–0.861) for the final model. The difference in AUC indicates that Delphi-based variable selection did not materially compromise predictive performance.

**Figure 4:**
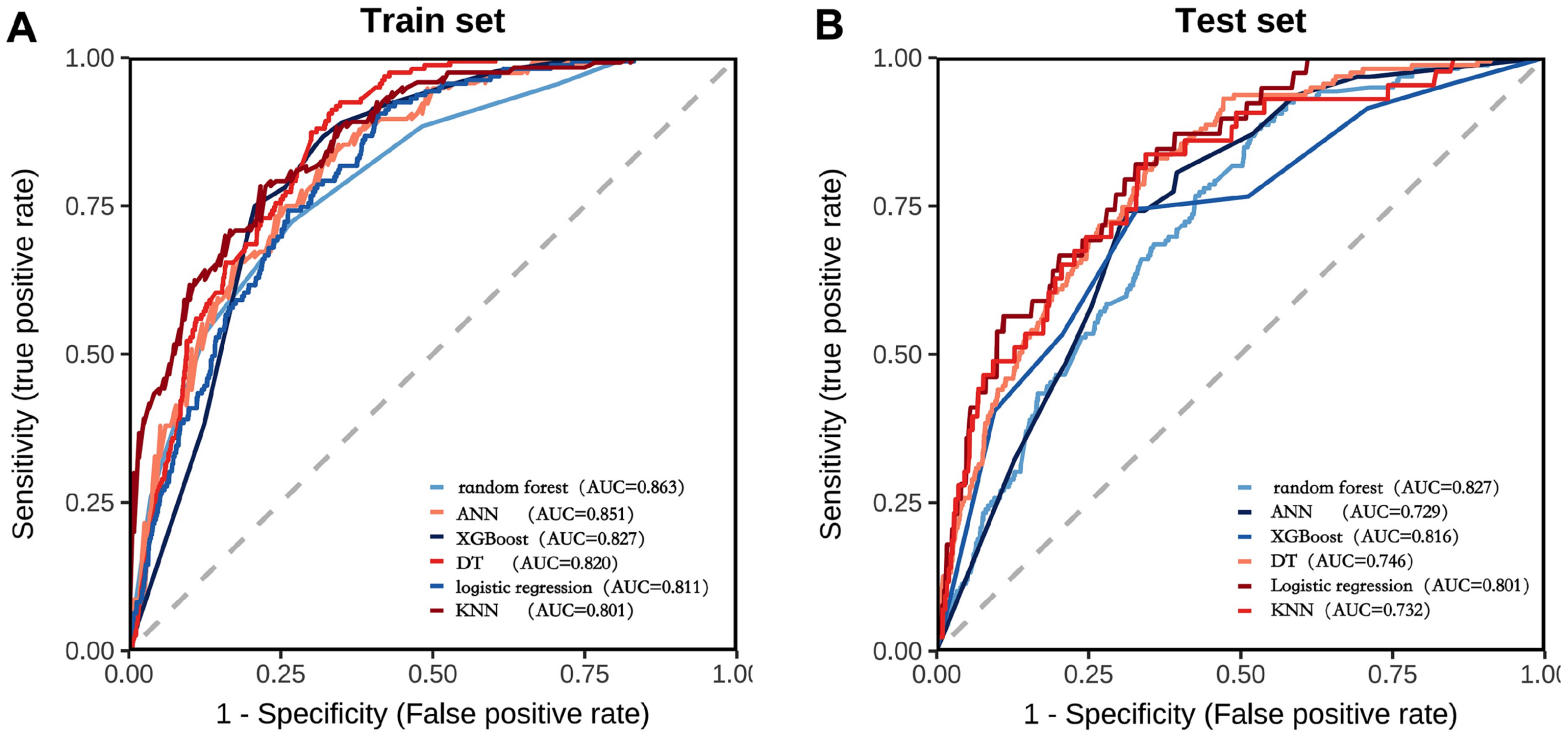
ROC comparison of six machine learning models: **(A)** ROC curves on the training set; **(B)** ROC curves on the internal test set. XGBoost achieved the highest test set AUC (0.816), followed by logistic regression (0.801). The dashed diagonal line represents a random classifier (AUC = 0.5). The x-axis shows 1 – specificity (false-positive rate), and the y-axis shows sensitivity (true-positive rate).

Based on the seven selected predictive variables, and after verifying that the training and test sets were comparable with no significant differences in key baseline characteristics (Supplementary Table S2), this study constructed six machine learning models: Random Forest, XGBoost, Logistic Regression, Artificial Neural Network, K-Nearest Neighbors, and Decision Tree. The optimal probability threshold derived from the Youden index in the internal test set was 0.41. Performance evaluation revealed that Logistic Regression and XGBoost models demonstrated optimal performance. The XGBoost model achieved an AUC of 0.816 (95% CI: 0.766–0.861) on the test set, with an accuracy of 0.733, recall of 0.760. The Logistic Regression model achieved an AUC of 0.801 (95% CI: 0.770–0.831) on the test set, with an accuracy of 0.711, recall of 0.723. The Random Forest model achieved a test set AUC of 0.827 (95% CI: 0.767–0.882), with an accuracy of 0.705, recall of 0.692, and specificity of 0.705. The AUC difference between training and test sets indicated good model generalization capability, as shown in Table 4 and Figure 4.

**Table 4:** Performance comparison of six machine learning models.

Model	Accuracy	Recall	Specificity	Training Set AUC	Test set AUC
Random forest	0.705	0.692	0.705	0.863 (95% CI: 0.823–0.893)	0.827 (95% CI: 0.767–0.882)
XGBoost	0.733	0.760	0.732	0.827 (95% CI: 0.785–0.850)	0.816 (95% CI: 0.766–0.861)
Logistic regression	0.711	0.723	0.710	0.811 (95% CI: 0.732–0.843)	0.801 (95% CI: 0.770–0.831)
Artificial neural network	0.621	0.692	0.620	0.851 (95% CI: 0.813–0.892)	0.729 (95% CI: 0.690–0.758)
KNN	0.672	0.745	0.671	0.801 (95% CI: 0.695–0.830)	0.732 (95% CI: 0.656–0.808)
Decision tree	0.701	0.710	0.701	0.820 (95% CI: 0.735–0.840)	0.746 (95% CI: 0.674–0.818)

To assess the stability of the six models under random undersampling, we performed five repeated runs for each model. As shown in Supplementary Table S4, the SD of AUC across the five runs were small, indicating that the performance estimates are robust and not due to a particular random draw. Random Forest, XGBoost, and Logistic Regression consistently outperformed the other three models. Furthermore, a sensitivity analysis using SMOTE (Supplementary Table S5) yielded similar AUC values for XGBoost (e.g., 0.812 ± 0.003 vs. 0.824 ± 0.011 for undersampling). Random undersampling performed slightly better than SMOTE in terms of both AUC and recall.

External validation was performed using the independent MIMIC-IV database (n = 4,932, of whom 116 (2.35%) had GNB-BSI). Baseline characteristics of the internal cohort and the external cohort are compared in Supplementary Table S6. Because PCT was not available in MIMIC-IV, we used the logistic regression model (which is less sensitive to missing predictors) with the six available variables (age, venous catheter, LOS, min_wbc, max_crp, max_neutrophil_percentage). When applied to the MIMIC-IV cohort, the model yielded an AUC of 0.744(95% CI: 0.700–0.787), with a recall of 0.647, an accuracy of 0.755 and specificity of 0.757 (Supplementary Figure S2, Supplementary Table S7).

As a sensitivity analysis, we retrained the model on the complete-case internal cohort (exclude cases where any one of the seven variables is missing) and validated it on MIMIC-IV, yielding an AUC of 0.725 (95% CI: 0.681–0.769), sensitivity 0.638, specificity 0.715, and accuracy 0.713 (Supplementary Figure S3). The comparable performance across the two missing-data handling approaches confirms the robustness of our primary imputation strategy.

**Figure 5:**
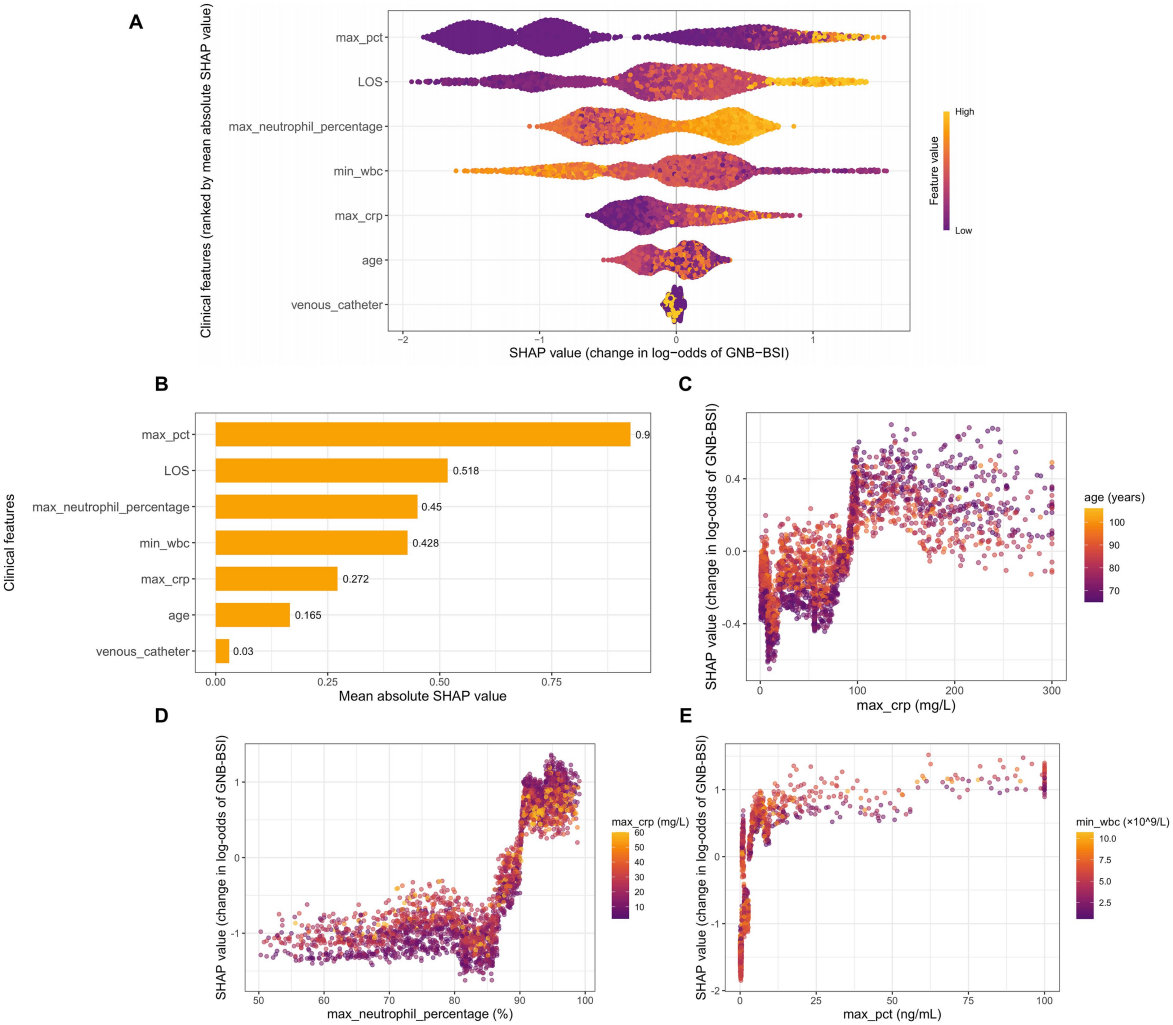
SHAP analysis for model interpretation. **(A)** Bee swarm plot of SHAP values for each feature. The horizontal axis shows the SHAP value (change in the log-odds of GNB-BSI). Features are ordered by mean absolute SHAP value. Red dots indicate high feature values, blue dots low feature values; **(B)** Feature importance bar plot. The horizontal axis shows the mean absolute SHAP value (importance). Numerical values are displayed on the bars. max_pct is the most important predictor; **(C–E)** SHAP dependence plots for max_crp **(C)**, max_neutrophil_percentage **(D)**, and max_pct **(E)**. The horizontal axis shows the measured value of the feature; the vertical axis shows the SHAP value (contribution to the prediction).

SHAP analysis results are shown in Figure 5. The top six variables by feature importance were: max_pct, LOS, max_neutrophil_percentage, min_wbc, max_crp, and age. The direction and magnitude of their contribution to model predictions were visually represented through SHAP value distributions. The SHAP dependency plots clearly depict the nonlinear relationship between continuous variables and predicted risk. Different patterns were observed across inflammatory markers: for example, the contribution of max_neutrophil_percentage to predicted risk increased substantially after exceeding a certain value, while the contributions of max_pct and max_crp tended to plateau beyond certain levels.

To further enhance clinical portability, we developed a nomogram based on the final logistic regression model (Supplementary Figure S5). This tool allows clinicians to estimate an individual patient’s risk without manual calculations, facilitating rapid bedside decision-making.

## 4 Discussion

Given the clinical challenges posed by the high occult nature and poor prognosis of GNB-BSI in the elderly, there is currently a lack of effective early prediction tools. Furthermore, previous studies have mostly relied on single-center or open databases (such as MIMIC), raising questions about their generalizability (28–33). To address this, this study successfully integrated multi-center big data with machine learning algorithms to construct an early risk prediction model for GNB-BSI in elderly patients.

A timeline-based schematic flowchart (Supplementary Figure S4) contrasts the traditional diagnostic pathway (48–72 h waiting for blood culture results) with the model-assisted pathway. When a non-blood GNB specimen tests positive, the model computes a risk score. If the score exceeds the chosen threshold (e.g., 0.41), the following actions are recommended (guided by local antimicrobial stewardship guidelines): obtain blood cultures immediately; initiate empirical broad-spectrum Gram-negative antibiotic therapy; intensify clinical and laboratory monitoring (e.g., serial PCT, CRP); and evaluate source control if a focus of infection is identifiable. For patients below the threshold, continued observation without immediate escalation is safe, supported by the model’s high negative predictive value (0.994). Clinicians may adjust the threshold locally to balance false positives and recall. As an illustration of a high-risk scenario, consider an 80-year-old patient with a venous catheter, CRP 120 mg/L, PCT 8 ng/mL, LOS 25 days, min_wbc 4.5 × 10^9^/L, and max_neutrophil_percentage 92%. The model predicts a GNB-BSI risk of 0.68, which exceeds the recommended threshold of 0.41. Accordingly, we suggest obtaining blood cultures, initiating empirical Gram-negative antibiotics, and considering ICU consultation. Conversely, a low-risk example would be a 72-year-old patient without a venous catheter, with CRP 30 mg/L, PCT 0.4 ng/mL, LOS 8 days, min_wbc 6.8 × 10^9^/L, and max_neutrophil_percentage 78%. The predicted risk is 0.12 (below the threshold), and continued observation without antibiotics is appropriate. The model’s strengths lie in its clinical utility and methodological robustness. The robustness of our model to the class imbalance handling strategy was confirmed by five-fold repeated random undersampling and a comparison with SMOTE, both of which yielded stable and consistent AUC estimates. For real-world deployment, the model can be embedded into hospital EHR systems as a decision support tool. The seven routinely captured variables automatically generate a risk score and SHAP breakdown upon a positive non-blood GNB result, helping clinicians understand high-risk flags and act with confidence.

Unlike prediction tools that attempt universal screening at hospital admission, our model is designed for application after the first detection of GNB in non-blood specimens. This targeted timing is based on the following considerations. First, the overall incidence of GNB-BSI is extremely low, making universal screening prone to identifying few true high-risk patients from a large negative population while generating numerous false-positive alerts that increase clinical burden. Second, once GNB is detected in non-blood specimens, the clinical scenario becomes more complex. This finding could signal an impending bloodstream infection, but it might also represent only local colonization or contamination that does not require antibiotic intervention. Clinicians thus face a genuine dilemma regarding whether to initiate empirical anti-infective therapy: overtreatment increases antibiotic exposure and antimicrobial resistance risk, while delayed treatment may miss the optimal window for intervention. Risk stratification in this specific context can more precisely address clinical needs. This well-defined application scenario ensures that the model serves as a decision support tool at a specific clinical time point, rather than a universal screening instrument. Methodologically, a three-stage feature selection process (LASSO regression, Boruta algorithm, and Delphi expert consultation) ensured variable criticality and interpretability, while undersampling techniques addressed class imbalance. Ultimately, the XGBoost and logistic regression models achieved AUC values of 0.816 and 0.801, respectively, demonstrating robust predictive performance.

Crucially, model interpretation using SHAP values overcomes the “black box” limitations of machine learning, enabling personalized decision support for clinicians.

Variable importance analysis further revealed the model’s biological implications. This study identified max_pct as one of the most critical predictive variables, aligning with findings by Oberhoffer et al. (34) who demonstrated PCT’s superiority over traditional inflammatory markers like CRP and WBC in distinguishing severe infections. This collectively confirms PCT’s outstanding value in infection risk stratification. SHAP analysis further demonstrated the positive contributions of markers such as max_crp and max_neutrophil_percentage (35, 36), quantitatively validating the core pathophysiological mechanism of systemic inflammatory response in GNB-BSI among the elderly. The negative correlation between min_wbc and risk provides crucial insights into the immune status of elderly GNB-BSI patients. This low indicator may not stem from insufficient bone marrow reserves but rather signifies a functional state of “immune paralysis” (37, 38). Its microscopic basis includes endotoxin tolerance phenomena such as impaired monocyte response function (e.g., reduced TNF-*α* production after LPS stimulation), indicating innate immune system failure and an inability to mount an effective response to pathogens. This profoundly reveals the underlying cause of high mortality in such patients. Furthermore, the positive correlation between LOS and age separately reveals independent risks associated with healthcare exposure and immune aging (39–41). Ultimately, venous_catheter, as the sole modifiable iatrogenic factor in the model, underscores the direct importance of rigorously implementing infection control strategies (42–44). All biological interpretations presented above are exploratory and based on statistical associations. They should not be overinterpreted as causal mechanisms, and future experimental or prospective studies are needed to validate these hypotheses.

SHAP dependency analysis further revealed that inflammatory markers represented by max_pct exhibit a common threshold pattern in predicting GNB-BSI in the elderly. The analysis showed that the predictive value of key inflammatory markers such as max_pct and max_crp does not increase linearly but exhibits a significant jump after exceeding specific thresholds (e.g., max_pct > 5 ng/mL). This observation aligns closely with the clinical findings of Yang et al. (45), confirming that inflammation levels above specific thresholds strongly indicate infection risk. Crucially, the model reveals that the contribution of these markers enters a plateau phase beyond a higher threshold (e.g., max_pct > 12.5 ng/mL), implying that their concentration changes become saturated in distinguishing risk beyond this point. This finding holds promise for simplifying clinical decision-making processes. However, caution is warranted when applying this universal model to specific populations, such as patients with hematologic disorders. Sun et al. noted (46) that PCT levels in such patients with BSI are generally low, predominantly concentrated below 0.5 ng/mL, rendering conventional diagnostic thresholds less effective. This suggests the potential need for more precise threshold values tailored to specific groups. This shared “threshold-plateau” kinetic pattern may reflect an “all-or-nothing” response characteristic of the immune-inflammatory system in elderly patients, providing crucial theoretical support for future development of individualized, simplified clinical decision rules.

The feature selection process effectively identified genuine biological signals. Although blood_transfusion positively correlates with risk (consistent with the TRIM effect (47–49)) in LASSO, it was excluded via the Delphi method due to its complex etiology and insufficient specificity (e.g., surgery, anemia, coagulation disorders), which could lead to misinterpretation as a predictor (50). The negative correlation with “community_infection” in LASSO likely reflects coding biases driven by healthcare policies (e.g., underreporting of catheter-associated infections (51)) rather than genuine biological effects. These cases underscore the irreplaceable value of expert consensus methods like the Delphi approach in filtering spurious statistical associations and enhancing clinical credibility within multicenter real-world data. Our sensitivity analysis confirmed that this exclusion not only did not compromise predictive performance but actually improved it (AUC increased from 0.805 to 0.816), supporting the prioritization of clinical interpretability while enhancing predictive accuracy. This hybrid approach offers a useful paradigm for developing clinically acceptable prediction tools. The model excluded traditional factors like “ICU admission” and “MDR organisms” challenging conventional wisdom yet aligning strongly with underlying pathophysiological mechanisms. Information about “ICU admission” is already captured by earlier physiological indicators such as inflammatory markers (52). More significantly, the exclusion of “MDR bacteria” strongly suggests a “virulence-resistance trade-off” among pathogens (53), where highly resistant strains may generally exhibit weaker pathogenicity than highly virulent susceptible strains (54). Thus, the latter, rather than the former, is more likely to drive early severe infection and inflammatory storms. While this hypothesis is biologically plausible and supported by some in studies, it remains controversial in clinical settings.

From a clinical implementation perspective, the choice between XGBoost and logistic regression should be guided by the specific priorities of the target setting. XGBoost offers superior model stability and a higher recall for detecting true GNB-BSI cases, making it preferable in high-stakes environments where missing a case carries severe consequences and where data quality may vary. Conversely, logistic regression provides a simpler, more transparent decision rule that can be readily implemented as a bedside scoring system or within electronic health records without specialized software, and its performance remains acceptable (AUC 0.801). Thus, rather than endorsing a single best model, we recommend that institutions select the model that best aligns with their clinical workflow, available technical support, and tolerance for false negatives. The low incidence of GNB-BSI (1.68%) leads to a low positive predictive value, raising concern about alert fatigue. However, in high-stakes infections, minimizing false negatives (missed cases) is far more critical than reducing false positives. The harm of a missed GNB-BSI outweighs false alarms, which are manageable via clinical assessment or blood culture. Hence, a model prioritizing recall (0.760) with a moderate false-positive rate is clinically defensible. Our model provides a probabilistic risk score to augment, not replace, clinical judgment; clinicians can override alerts inconsistent with their assessment. To mitigate alert fatigue, future implementation could use risk scores with confidence intervals and require active interpretation. Thus, the model serves as a safety net rather than a diagnostic arbiter, aligning with “better safe than sorry” in elderly patients at risk of GNB-BSI.

External validation using MIMIC-IV revealed slightly lower performance compared to the internal test set, mainly due to the absence of PCT. Nonetheless, the model using six predictors still achieved a moderate AUC of 0.744 (95% CI: 0.700–0.787), with a recall of 0.647. Notably, the internal Sichuan cohort and the external MIMIC-IV cohort differ substantially in race/ethnicity, healthcare systems, and clinical practices, yet our model achieved acceptable performance on the extremely imbalanced database. This supports the robustness and generalizability of our model beyond the original development setting. These results underscore the importance of PCT for optimal performance and highlight that external validation in independent datasets is essential to avoid overoptimistic estimates. Future work should validate the full 7-predictor model in cohorts where PCT is routinely measured.

This study has several limitations. First, external validation was performed using MIMIC-IV, but due to the unavailability of PCT, only a six-predictor version could be validated; the generalizability of the full seven-predictor model thus awaits further testing in datasets containing PCT. Second, the retrospective design precludes causal inference, thus, all associations should be interpreted as predictive rather than causal, and that unmeasured confounding factors (e.g., treatment interventions, timing of laboratory measurements, inter-center heterogeneity) may not be fully addressed. Selection bias may arise from including only clinically indicated cultures, skewing the cohort toward sicker patients. Thus, the model should be used only for patients with non-blood specimen cultures. Third, while the Delphi method enhanced clinical interpretability, it inherently introduces subjective judgment in variable selection, which may exclude statistically informative predictors that could be valuable in diverse populations, but its impact on model performance was benign or beneficial in this study. Fourth, in our data extraction we defined predictor values as the maximum or minimum of each laboratory test recorded up to the date of GNB detection, without extracting precise time stamps. Because all measurements were obtained during the same hospitalization episode, any minor temporal misalignment between the measurement time and the actual infection onset is unlikely to have a meaningful impact on the overall results. Future studies could further refine the prediction window by collecting time-stamped data. The model assumes that laboratory tests are performed at least once during the pre-detection period. In settings with very low testing frequency, the maximum or minimum values may not capture the true physiological extremes, which could affect performance. However, given that elderly inpatients with suspected infection typically receive daily or every-other-day monitoring, this limitation is likely minimal. Fifth, the threshold effects observed in SHAP analysis are exploratory and require prospective validation before they can inform clinical decision-making. Finally, median imputation may underestimate variance, though missing rates were low.

## 5 Conclusion

In summary, this study developed a predictive model with satisfactory performance for GNB-BSI risk in elderly patients. This tool holds promise for assisting clinicians in achieving earlier, more precise interventions, thereby improving outcomes for elderly patients with infections.

## Data Availability

The raw data supporting the conclusions of this article will be made available by the authors, without undue reservation.

## Glossary

GNB-BSI: Gram-Negative Bacterial Bloodstream Infection
BSI: Bloodstream Infection
GNB: Gram-Negative Bacteria
GPB: Gram-Positive Bacteria
ICU: Intensive Care Unit
LOS: Length of Hospital Stay
MDR: Multidrug-Resistant
CRP: C-reactive Protein
PCT: Procalcitonin
WBC: White Blood Cell (Count)
CLSI: Clinical and Laboratory Standards Institute
SD: Standard deviation
SMOTE: Synthetic Minority Oversampling Technique
LASSO: Least Absolute Shrinkage and Selection Operator
AUC: Area Under the (ROC) Curve
ROC: Receiver Operating Characteristic
XGBoost: eXtreme Gradient Boosting
SHAP: SHapley Additive exPlanations
CV: Coefficient of Variation
Kendall’s W: Kendall’s Coefficient of Concordance
COPD: Chronic Obstructive Pulmonary Disease
max_wbc: maximum white blood cell count
min_wbc: minimum white blood cell count
max_platelet: maximum platelet count
max_neutrophil_percentage: maximum neutrophil percentage
max_crp: maximum C-reactive protein level
max_pct: maximum procalcitonin level
max_total_protein: maximum total protein level
min_albumin: minimum albumin level
