# Supplementary material for: Predicting Gram-negative bloodstream infection in elderly patients after isolation of GNB from non-blood specimens: a machine learning-based tool
Source: Front Med (Lausanne). 2026 Jun 16;13:1819369. doi: 10.3389/fmed.2026.1819369 (PMC13314445; doi:10.3389/fmed.2026.1819369)
Supplement: Supplementary file 1 [file Data_Sheet_1.docx]

Table S1 Diagnostic Criteria for GNB-BSI

| **Infection Type** | Classification | **Diagnostic Criteria** |
| --- | --- | --- |
| **I. Vascular Catheter-Related Infection​** | **Clinical Diagnosis​** | Meeting any one of the following criteria:  1. Local signs: Purulent drainage from the venous puncture site, or diffuse erythema (cellulitis).  2. Catheter tract symptoms: Painful diffuse erythema along the subcutaneous tract of the catheter (excluding physical/chemical causes).  3. Post-intervention fever: Fever (>38°C) with local tenderness after a vascular intervention procedure, with no other explanation. |
|  | **Etiological Diagnosis** | **Meeting any one of the following criteria on the basis of clinical diagnosis:**  **• Significant GNB isolated from culture of the catheter tip.**  **• Significant GNB isolated from blood culture.** |
|  | **Notes​** | 1. Catheter tip culture: The catheter tip (5 cm segment) is rolled on a blood agar plate; growth of ≥15 CFU/plate is considered positive.  2. Quantitative blood culture: Quantitative culture from the puncture site with a bacterial count ≥100 CFU/mL, or 4-10 times higher than the contralateral side, or the same organism isolated from the contralateral culture. |
| **II. Sepsis​** | **Clinical Diagnosis​** | **Fever (>38°C) or hypothermia (<36°C), which may be accompanied by chills, PLUS any one of the following:**  **1. A clear portal of entry or metastatic focus of infection.**  **2. Systemic toxic symptoms without a clear infection site.**  **3. Rash, petechiae, hepatosplenomegaly, neutrophilia with a left shift, unexplained by other causes.**  **4. Systolic blood pressure below 90 mmHg or a decrease of more than 40 mmHg from baseline.** |
|  | **Etiological Diagnosis​** | **Meeting any one of the following criteria on the basis of clinical diagnosis:**  **1. Isolation of GNB from blood culture.**  **2. Detection of Gram-negative bacterial antigens in the blood.** |
|  | **Notes​** | 1. Recurrent infection: A new positive blood culture (excluding contaminants) after admission counts as a separate hospital-acquired infection.  2. Common skin commensals (e.g., Coagulase-negative staphylococci): Require positivity in two or more blood cultures drawn at different times.  3. Antigen detection: Must be consistent with clinical symptoms and signs.  4. Catheter-related sepsis is categorized here.  5. Polymicrobial infection: Diagnosis requires growth of multiple organisms in blood culture, after contamination is ruled out. |
| **III. Transfusion-Transmitted Infection** | **Clinical Diagnosis** | **Meeting ALL of the following conditions:**  **1. Temporal relationship: The time from transfusion to onset of illness or appearance of immunological markers exceeds the average incubation period of the pathogen.**  **2. Pre-transfusion negativity: The recipient had no evidence of this infection prior to transfusion, and immunological markers were negative.**  **3. Donor evidence: Confirmation of infectious agents (pathogen, immunological markers, pathogen DNA/RNA positivity, etc.) in the donor's blood.** |
|  | **Etiological Diagnosis​** | **Meeting any one of the following criteria on the basis of clinical diagnosis:**  **1. Identification of the Gram-negative bacterium in the blood.**  **2. Positive detection of specific Gram-negative bacterial antigens in the blood, OR diagnostic levels of IgM antibody, OR a four-fold increase in IgG antibody titers in paired serum samples.**  **3. Finding inclusion bodies in tissue or body fluid smears.**  **4. Confirmation by pathological biopsy.** |
|  | **Notes​** | 1. Clinical presentation: The patient may have symptoms and signs, or only immunological changes.  2. HIV exception: A positive HIV antibody test within 6 months after transfusion can serve as preliminary evidence, but requires confirmatory testing. |

Table S2 Comparison of baseline characteristics between the training and test sets

| Variable | Training_Set(n=7716) | Test_Set(n=1930) | Statistic | P_value |
| --- | --- | --- | --- | --- |
| gender-male(%) | 4,949 (64.14%) | 1,217 (63.06%) | 0.738* | 0.390 |
| age(years) | 79.00 [71.00, 88.00] | 79.00 [71.00, 88.00] | 7432821.000^†^ | 0.905 |
| LOS(days) | 21.00 [12.00, 29.00] | 21.00 [12.00, 28.00] | 7528985.500^†^ | 0.448 |
| community_infection(%) | 1,843 (23.89%) | 436 (22.59%) | 1.363* | 0.243 |
| icu_admission(%) | 3,120 (40.44%) | 772 (40.00%) | 0.104* | 0.747 |
| blood_transfusion(%) | 2,476 (32.09%) | 630 (32.64%) | 0.192* | 0.661 |
| urinary_catheter(%) | 2,907 (37.67%) | 716 (37.10%) | 0.195* | 0.659 |
| venous_catheter(%) | 2,203 (28.55%) | 577 (29.90%) | 1.297* | 0.255 |
| mechanical_ventilation(%) | 1,864 (24.16%) | 508 (26.32%) | 3.782* | 0.052 |
| tracheostomy(%) | 636 (8.24%) | 175 (9.07%) | 1.259* | 0.262 |
| hypertension(%) | 4,256 (55.16%) | 1,071 (55.49%) | 0.057* | 0.812 |
| diabetes(%) | 2,375 (30.78%) | 623 (32.28%) | 1.552* | 0.213 |
| copd(%) | 1,617 (20.96%) | 370 (19.17%) | 2.901* | 0.089 |
| liver_failure(%) | 152 (1.97%) | 45 (2.33%) | 0.837* | 0.360 |
| renal_failure(%) | 661 (8.57%) | 148 (7.67%) | 1.506* | 0.220 |
| heart_failure(%) | 994 (12.88%) | 259 (13.42%) | 0.348* | 0.555 |
| respiratory_failure(%) | 1,731 (22.43%) | 464 (24.04%) | 2.179* | 0.140 |
| surgery(%) | 5,774 (74.83%) | 1,429 (74.04%) | 0.469* | 0.494 |
| max_wbc(×10^9^/L) | 10.83 [7.77, 14.05] | 10.83 [7.91, 14.02] | 7532646.000^†^ | 0.428 |
| min_wbc(×10^9^/L) | 5.54 [4.44, 6.59] | 5.54 [4.47, 6.66] | 7544750.500^†^ | 0.366 |
| max_neutrophil_rate(%) | 85.80 [76.30, 92.30] | 85.80 [76.00, 92.40] | 7416409.500^†^ | 0.787 |
| max_crp(mg/L) | 55.89 [9.88, 97.04] | 55.89 [10.85, 96.63] | 7464508.500^†^ | 0.865 |
| max_pct(ng/mL) | 1.36 [0.41, 4.45] | 1.36 [0.46, 4.45] | 7517804.500^†^ | 0.509 |
| max_platelet(×10^9^/L) | 215.00 [154.00, 290.00] | 215.00 [153.00, 279.00] | 7314584.500^†^ | 0.230 |
| max_total_protein(g/L) | 67.20 [62.90, 71.90] | 67.20 [62.60, 71.70] | 7338163.000^†^ | 0.324 |
| min_albumin(g/L) | 53.70 [37.10, 56.70] | 53.70 [38.30, 56.20] | 7444803.000^†^ | 0.992 |
| mdr_bacteria(%) | 2,169 (28.11%) | 557 (28.86%) | 0.392* | 0.531 |
| bacteria_class (%) |  |  | 3.020* | 0.389 |
| others | 458 (5.94%) | 121 (6.27%) |  |  |
| bacteria_enterobacteriaceae | 4,821 (62.48%) | 1,176 (60.93%) |  |  |
| bacteria_pseudomonas_aeruginosa | 1,510 (19.57%) | 376 (19.48%) |  |  |
| bacteria_acinetobacter_baumannii | 927 (12.01%) | 257 (13.32%) |  |  |

†Wilcoxon rank-sum test.

*Pearson's chi-squared test.

Table S3 Missing data proportions for candidate variables

| Variable | Missing cases (n) | Proportion of missing values |
| --- | --- | --- |
| gender-male | 0 | 0 |
| age | 0 | 0 |
| LOS | 0 | 0 |
| community_infection | 0 | 0 |
| icu_admission | 0 | 0 |
| blood_transfusion | 0 | 0 |
| urinary_catheter | 0 | 0 |
| venous_catheter | 0 | 0 |
| mechanical_ventilation | 0 | 0 |
| tracheostomy | 0 | 0 |
| hypertension | 0 | 0 |
| diabetes | 0 | 0 |
| copd | 0 | 0 |
| liver_failure | 0 | 0 |
| renal_failure | 0 | 0 |
| heart_failure | 0 | 0 |
| respiratory_failure | 0 | 0 |
| surgery | 0 | 0 |
| max_wbc | 806 | 8.36% |
| min_wbc | 806 | 8.36% |
| max_neutrophil_rate | 231 | 2.39% |
| max_crp | 631 | 6.54% |
| max_pct | 1053 | 10.92% |
| max_platelet | 808 | 8.38% |
| max_total_protein | 769 | 7.97% |
| min_albumin | 763 | 7.91% |
| mdr_bacteria | 0 | 0 |
| bacteria_class | 0 | 0 |
| others | 0 | 0 |
| bacteria_enterobacteriaceae | 0 | 0 |
| bacteria_pseudomonas_aeruginosa | 0 | 0 |
| bacteria_acinetobacter_baumannii | 0 | 0 |

Table S4 Performance stability of six machine learning models across five repeated random undersampling runs.

| Model | Run 1 | Run 2 | Run 3 | Run 4 | Run 5 | AUC (mean ± SD) | Original AUC(single run) |
| --- | --- | --- | --- | --- | --- | --- | --- |
| Random Forest | 0.802 | 0.791 | 0.813 | 0.815 | 0.799 | 0.804 ± 0.010 | 0.827 |
| XGBoost | 0.829 | 0.840 | 0.816 | 0.820 | 0.815 | 0.824 ± 0.011 | 0.816 |
| Logistic Regression | 0.816 | 0.826 | 0.808 | 0.813 | 0.785 | 0.809 ± 0.016 | 0.801 |
| Artificial Neural Network | 0.722 | 0.752 | 0.711 | 0.732 | 0.737 | 0.731 ± 0.016 | 0.729 |
| KNN | 0.783 | 0.773 | 0.744 | 0.729 | 0.756 | 0.757 ± 0.022 | 0.732 |
| Decision Tree | 0.764 | 0.742 | 0.720 | 0.722 | 0.690 | 0.728 ± 0.028 | 0.746 |

Table S5 Comparison of random undersampling and SMOTE for handling class imbalance (XGBoost model, 5 repeated runs).

| Imbalance handling method | Accuracy (single run) | Recall (single run) | Specificity (single run) | Test set AUC (mean ± SD) | AUC range |
| --- | --- | --- | --- | --- | --- |
| Random undersampling | 0.733 | 0.760 | 0.732 | 0.824 ± 0.011 | 0.815 – 0.840 |
| SMOTE | 0.731 | 0.727 | 0.731 | 0.812 ± 0.003 | 0.810 – 0.815 |

Table S6 Comparison of baseline characteristics between the internal cohort and the external MIMIC‑IV validation cohort.

| Variable | internal cohort(n=9646) | external cohort(n=4932) | Statistic | P_value |
| --- | --- | --- | --- | --- |
| age(years) | 79.48±9.59 | 77.30±7.89 | 12.083^†^ | <0.001 |
| LOS(days) | 22.29±13.60 | 16.49±12.70 | 29.452^†^ | <0.001 |
| venous_catheter(%) | 2780(28.82%) | 1180(23.93%) | 39.525* | <0.001 |
| min_wbc(×10^9^/L) | 5.58±1.83 | 6.11±2.06 | -16.067^†^ | <0.001 |
| max_neutrophil_rate(%) | 83.32±10.95 | 83.76±7.41 | 6.352^†^ | <0.001 |
| max_crp(mg/L) | 70.30±67.72 | 61.37±31.60 | -8.523^†^ | <0.001 |
| GNB-BSI | 159(1.65%) | 116(2.35%) | 8.735* | 0.003 |

Data are presented as mean ± SD for continuous variables and as count (percentage) for categorical variables. †Wilcoxon rank-sum test. *Pearson's chi-squared test.

Table S7 External validation performance in the MIMIC-IV cohort (logistic regression model, 6 predictors) .

| Metric | Value |
| --- | --- |
| Sample size, n | 4932 |
| GNB-BSI positive, n (%) | 116 (2.35) |
| AUC | 0.744(95% CI: 0.700-0.787) |
| Sensitivity (Recall) | 0.647 |
| Specificity | 0.755 |
| Accuracy | 0.757 |

Notes: External validation was performed using the MIMIC-IV database. Because PCT was not available in MIMIC database, the model included six predictors: age, venous catheter, LOS, min_wbc, max_crp, and max_neutrophil_rate.
