# Supplementary material for: Predicting Gram-negative bloodstream infection in elderly patients after isolation of GNB from non-blood specimens: a machine learning-based tool
Source: Front Med (Lausanne). 2026 Jun 16;13:1819369. doi: 10.3389/fmed.2026.1819369 (PMC13314445; doi:10.3389/fmed.2026.1819369)
Supplement: Supplementary file 2 [file Supplementary_file_1.docx]

##### Delphi expert consultation questionnaire (first round)

Dear expert:

Hello! We are currently conducting a study on the development of a risk prediction model for Gram-negative bacterial bloodstream infection (GNB-BSI) in elderly patients. Based on preliminary real-world clinical data, we have preliminarily screened the following predictive variables using LASSO regression and the Boruta algorithm. To ensure the clinical relevance and practicality of the model, we sincerely invite you to serve as a consulting expert to evaluate the importance of these variables. Your professional judgment is crucial to this study. We sincerely appreciate your support and contribution!

1. Basic Expert Information

- Years of service: □ 5-10 years □ 11-20 years □>20 years
- Your technical title: □Chief Physician □Associate Chief Physician □Attending Physician
- Your professional field: □ Infectious Diseases Department □ Critical Care Medicine Department □ Geriatrics Department □ Respiratory and Critical Care Medicine Department □ Others _____

1. Evaluation of the importance of predictive variables

- Research Background: This study aims to develop a machine learning model to predict the risk of subsequent GNB-BSI in elderly hospitalized patients following the first detection of Gram-negative bacteria in any clinical specimen (e.g., sputum, urine). The model utilizes clinical and laboratory indicators that are readily available at or before that decision point.
- Guidelines for Filling: Based on clinical experience, assess the importance of each variable for predicting subsequent GNB-BSI risk after Gram-negative bacteria are first detected in any specimen in elderly hospitalized patients using a 9-point Likert scale (1=very unimportant, 9=very important). To standardize the scoring criteria, please score according to the following definitions:

| Scoring range | Importance level | Core Meaning |
| --- | --- | --- |
| 1-3 points | Unimportant | The variable has no or only minimal contribution to the prediction of GNB-BSI risk. |
| 4-6 points | Moderately Important | The variable has some effect on the prediction, but its importance is uncertain or controversial. |
| 7-9 points | Important | This variable is a clear and important indicator to predict the risk of GNB-BSI. |

- Consensus criterion: If ≥75% of experts rate a variable within the 7-9 score range (i.e., the 'important' interval), consensus is reached on the variable's importance.
- Algorithm result reference: The "LASSO coefficient" and "Boruta importance" columns in the table demonstrate the objective screening results of the preliminary machine learning algorithm for your scoring reference. The final variable importance should still be determined by your clinical professional judgment.
- Other suggestions: Add a "Suggestions" column at the end of the sheet to provide feedback on variable definitions or additions.

| **variable classes** | **predictive variable** | **Variable Definition** | **LASSO coefficient** | **Boruta Importance (Rank)** | **Importance score (1–9)** | **Comments/Suggestions** |
| --- | --- | --- | --- | --- | --- | --- |
| **Basic features** | age | Actual age at admission | - | 4.49 (12) | □1 □2 □3 □4 □5 □6 □7 □8 □9 |  |
|  | LOS | length of hospital stay | 0.0088 | 2.94 (14) | □1 □2 □3 □4 □5 □6 □7 □8 □9 |  |
| **complication** | copd | The admission diagnosis included chronic obstructive pulmonary disease (COPD). | - | 3.03 (13) | □1 □2 □3 □4 □5 □6 □7 □8 □9 |  |
|  | liver_failure | meeting the diagnostic criteria for liver failure at admission or during hospitalization | 0.0444 | -2.99 (Rejected) | □1 □2 □3 □4 □5 □6 □7 □8 □9 |  |
|  | respiratory_failure | Respiratory insufficiency requiring oxygen therapy or mechanical ventilation support during hospitalization | - | 4.74 (11) | □1 □2 □3 □4 □5 □6 □7 □8 □9 |  |
| **Infection and Treatment** | community_infection | Presence of community-acquired infection at admission | -0.1973 | 1.15 (Rejected) | □1 □2 □3 □4 □5 □6 □7 □8 □9 |  |
|  | surgery | Received any surgical procedure during hospitalization | - | 7.92 (6) | □1 □2 □3 □4 □5 □6 □7 □8 □9 |  |
|  | blood_transfusion | Received whole blood or component blood transfusion during hospitalization | 0.2965 | -1.39 (Rejected) | □1 □2 □3 □4 □5 □6 □7 □8 □9 |  |
| **Hospital Management and Operations** | icu_admission | Whether transferred to the intensive care unit (ICU) during hospitalization | - | 8.11(5) | □1 □2 □3 □4 □5 □6 □7 □8 □9 |  |
|  | venous_catheter | Central or peripheral venous catheterization during hospitalization | 0.0972 | 4.91 (10) | □1 □2 □3 □4 □5 □6 □7 □8 □9 |  |
|  | urinary_catheter | Indwelling urinary catheterization during hospitalization | - | 6.41 (8) | □1 □2 □3 □4 □5 □6 □7 □8 □9 |  |
| **Laboratory indicators (peak values)** | max_pct | The highest procalcitonin level detected during multiple tests while hospitalized | 0.0119 | 11.92 (4) | □1 □2 □3 □4 □5 □6 □7 □8 □9 |  |
|  | max_crp | The highest CRP value detected during multiple tests during hospitalization | 0.0037 | 14.31 (2) | □1 □2 □3 □4 □5 □6 □7 □8 □9 |  |
|  | max_neutrophil_rate | The highest percentage of neutrophils detected during multiple tests while hospitalized | 0.0283 | 14.48 (1) | □1 □2 □3 □4 □5 □6 □7 □8 □9 |  |
|  | max_wbc | The highest white blood cell count recorded during multiple tests while hospitalized | - | 12.7 (3) | □1 □2 □3 □4 □5 □6 □7 □8 □9 |  |
|  | max_platelet | The highest platelet count recorded during multiple tests while hospitalized | - | 5.09 (9) | □1 □2 □3 □4 □5 □6 □7 □8 □9 |  |
| **Laboratory indicators (baseline values)** | min_wbc | The lowest white blood cell count measured multiple times during hospitalization | -0.0647 | 2.38 (Tentative) | □1 □2 □3 □4 □5 □6 □7 □8 □9 |  |
|  | min_albumin | The lowest albumin level detected during multiple tests while hospitalized | - | 7.25 (7) | □1 □2 □3 □4 □5 □6 □7 □8 □9 |  |

* LASSO coefficient: positive values indicate positive correlation, negative values indicate negative correlation. “-” indicates not selected by LASSO.

* Boruta Importance: The higher the score, the more important the variable is considered by the algorithm. (Ranking) is a descending order ranking based on meanImp (1 being the most important).Rejected indicates the variable is deemed unimportant by the algorithm. Tentative indicates the result is tentative.

* The variables ultimately included in the Delphi process were the union of LASSO and Boruta results (i.e., variables present in either algorithm's output).

1. Expert self-assessment of authority
2. Judgment basis (Ca) self-assessment

| **judgment basis** | **Degree of impact** | | |
| --- | --- | --- | --- |
|  | **Significant** | **Moderate** | **Weak** |
| A. Practical Experience | (0.5)□ | (0.4)□ | (0.3)□ |
| B. Theoretical Analysis | (0.3)□ | (0.2)□ | (0.1)□ |
| C. Literature Analysis | (0.1)□ | (0.1)□ | (0.1)□ |
| D. Intuitive Feeling | (0.1)□ | (0.1)□ | (0.1)□ |

Please rate how much each criterion influenced your decision when answering this questionnaire (mark "√" in the corresponding cell)

1. Self-assessment of familiarity (Cs)

Please select the most appropriate option from the following based on your familiarity with the question 'Risk prediction of GNB-BSI in elderly patients'.

| **Level of Familiarity** | **Very Familiar** | **Relatively Familiar** | **Moderately Familiar** | **Slightly Familiar** | **Unfamiliar** |
| --- | --- | --- | --- | --- | --- |
| **assignment** | 1.0 | **0.8** | **0.6** | **0.4** | **0.2** |
| **Your choice** | □ | □ | □ | □ | □ |

We sincerely appreciate your support and cooperation. The information you provided is of critical importance to this study.
