# Supplementary material for: Predicting Gram-negative bloodstream infection in elderly patients after isolation of GNB from non-blood specimens: a machine learning-based tool
Source: Front Med (Lausanne). 2026 Jun 16;13:1819369. doi: 10.3389/fmed.2026.1819369 (PMC13314445; doi:10.3389/fmed.2026.1819369)
Supplement: Supplementary file 3 [file Supplementary_file_2.docx]

##### Delphi expert consultation questionnaire (second round)

Dear expert:

We sincerely appreciate your participation in the first expert consultation and your valuable feedback despite your busy schedule. After conducting statistical analysis of the first round's results, we have developed this updated questionnaire. The primary objective of this round is to anonymously share the aggregate statistical results with you, providing an opportunity for reassessment to help us further consolidate consensus and ultimately determine the core variables of the predictive model.

1. Re-evaluation Table of Predictive Variable Importance

- Feedback note: This round of the questionnaire includes a new column titled 'First Round Statistical Results,' which anonymously displays the mean and coefficient of variation of all expert scores from the previous round for your reference during this evaluation.
- Study Context Reminder: The model aims to predict the risk of subsequent GNB-BSI after Gram-negative bacteria are first detected in any clinical specimen, not at the time of admission.
- Scoring Guidelines Reminder: Please score using the same 9-point scale, where: 1-3 = Unimportant, 4-6 = Moderately Important, and 7-9 = Important. Consensus is defined as ≥75% of ratings falling within the 7-9 range (“Important”).
- Please refer to these statistical results and, in conjunction with your own clinical experience, re-evaluate the importance of each variable (1-9 points). Your reassessment is crucial for advancing from a 'preliminary consensus' to a 'high consensus.'
- Please pay special attention to variables with higher CV values (>25%) in the first round, as these variables showed considerable divergence in expert opinions, and your current judgment will play a critical role.

| **variable classes** | **predictive variable** | **Variable Definition** | **LASSO coefficient** | **Boruta Importance (Rank)** | **Importance score (1–9)** | **First round of statistical results** | | **Comments/Suggestions** |
| --- | --- | --- | --- | --- | --- | --- | --- | --- |
|  |  |  |  |  |  | **Mean ± SD** | **CV** |  |
| **Basic features** | age | Actual age at admission | - | 4.49 (12) | □1 □2 □3 □4 □5 □6 □7 □8 □9 | 7.2±1.7 | 0.241 |  |
|  | LOS | length of hospital stay | 0.0088 | 2.94 (14) | □1 □2 □3 □4 □5 □6 □7 □8 □9 | 7.6±1.3 | 0.166 |  |
| **complication** | copd | The admission diagnosis included chronic obstructive pulmonary disease (COPD). | - | 3.03 (13) | □1 □2 □3 □4 □5 □6 □7 □8 □9 | 3.8±2.4 | 0.633 |  |
|  | liver_failure | meeting the diagnostic criteria for liver failure at admission or during hospitalization | 0.0444 | -2.99 (Rejected) | □1 □2 □3 □4 □5 □6 □7 □8 □9 | 4.7±2.3 | 0.480 |  |
|  | respiratory_failure | Respiratory insufficiency requiring oxygen therapy or mechanical ventilation support during hospitalization | - | 4.74 (11) | □1 □2 □3 □4 □5 □6 □7 □8 □9 | 3.8±2.4 | 0.636 |  |
| **Infection and Treatment** | community_infection | Presence of community-acquired infection at admission | -0.1973 | 1.15 (Rejected) | □1 □2 □3 □4 □5 □6 □7 □8 □9 | 3.4±2.0 | 0.598 |  |
|  | surgery | Received any surgical procedure during hospitalization | - | 7.92 (6) | □1 □2 □3 □4 □5 □6 □7 □8 □9 | 3.8±2.6 | 0.670 |  |
|  | blood_transfusion | Received whole blood or component blood transfusion during hospitalization | 0.2965 | -1.39 (Rejected) | □1 □2 □3 □4 □5 □6 □7 □8 □9 | 6.9±1.7 | 0.246 |  |
| **Hospital Management and Operations** | icu_admission | Whether transferred to the intensive care unit (ICU) during hospitalization | - | 8.11(5) | □1 □2 □3 □4 □5 □6 □7 □8 □9 | 7.0±1.2 | 0.175 |  |
|  | venous_catheter | Central or peripheral venous catheterization during hospitalization | 0.0972 | 4.91 (10) | □1 □2 □3 □4 □5 □6 □7 □8 □9 | 7.2±1.7 | 0.234 |  |
|  | urinary_catheter | Indwelling urinary catheterization during hospitalization | - | 6.41 (8) | □1 □2 □3 □4 □5 □6 □7 □8 □9 | 7.0±1.4 | 0.202 |  |
| **Laboratory indicators (peak values)** | max_pct | The highest procalcitonin level detected during multiple tests while hospitalized | 0.0119 | 11.92 (4) | □1 □2 □3 □4 □5 □6 □7 □8 □9 | 7.5±1.8 | 0.236 |  |
|  | max_crp | The highest CRP value detected during multiple tests during hospitalization | 0.0037 | 14.31 (2) | □1 □2 □3 □4 □5 □6 □7 □8 □9 | 7.1±1.8 | 0.248 |  |
|  | max_neutrophil_rate | The highest percentage of neutrophils detected during multiple tests while hospitalized | 0.0283 | 14.48 (1) | □1 □2 □3 □4 □5 □6 □7 □8 □9 | 7.2±1.7 | 0.241 |  |
|  | max_wbc | The highest white blood cell count recorded during multiple tests while hospitalized | - | 12.7 (3) | □1 □2 □3 □4 □5 □6 □7 □8 □9 | 7.0±1.5 | 0.218 |  |
|  | max_platelet | The highest platelet count recorded during multiple tests while hospitalized | - | 5.09 (9) | □1 □2 □3 □4 □5 □6 □7 □8 □9 | 4.0±2.5 | 0.637 |  |
| **Laboratory indicators (baseline values)** | min_wbc | The lowest white blood cell count measured multiple times during hospitalization | -0.0647 | 2.38 (Tentative) | □1 □2 □3 □4 □5 □6 □7 □8 □9 | 7.0±1.7 | 0.247 |  |
|  | min_albumin | The lowest albumin level detected during multiple tests while hospitalized | - | 7.25 (7) | □1 □2 □3 □4 □5 □6 □7 □8 □9 | 7.1±1.3 | 0.187 |  |

We sincerely appreciate your outstanding contributions and strong support! Your insights were pivotal to the success of this study.
