# Supplementary material for: Predicting Gram-negative bloodstream infection in elderly patients after isolation of GNB from non-blood specimens: a machine learning-based tool
Source: Front Med (Lausanne). 2026 Jun 16;13:1819369. doi: 10.3389/fmed.2026.1819369 (PMC13314445; doi:10.3389/fmed.2026.1819369)
Supplement: Supplementary file 4 [file Supplementary_file_3.docx]

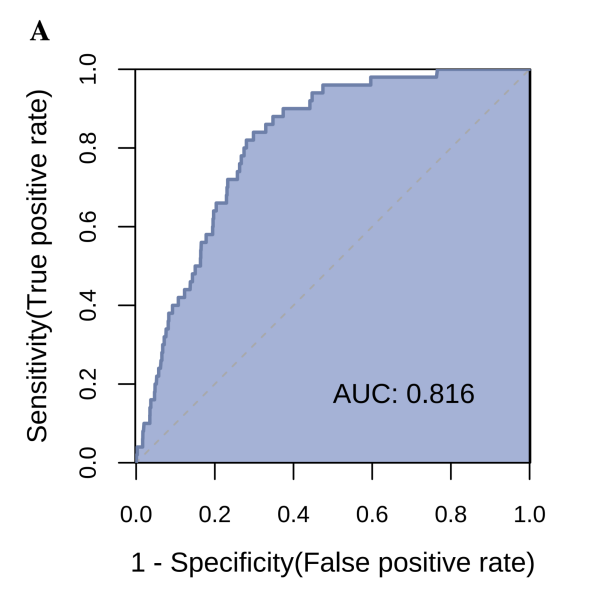

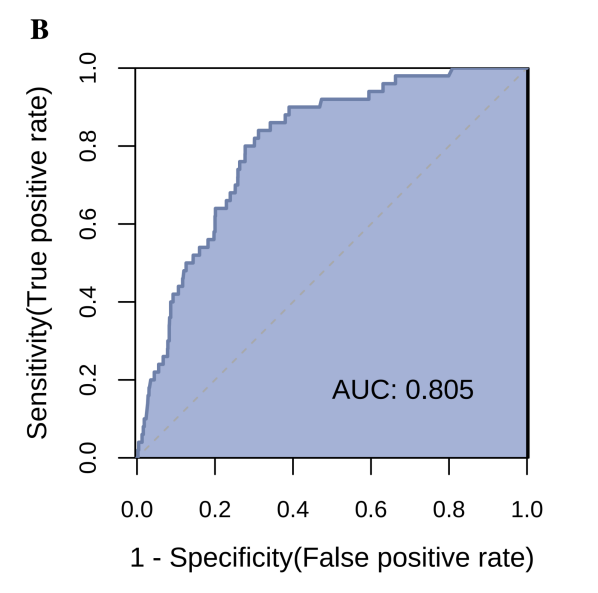


Figure S1. ROC curves comparing XGBoost models with and without Delphi-based variable selection. (A) ROC curve for the final XGBoost model after Delphi-based variable selection (n=7 variables). (B) ROC curve for the XGBoost model based on variables selected by LASSO regression and the Boruta algorithm, without Delphi-based exclusion (n=18 variables). The diagonal dashed line indicates the line of no discrimination. The AUC values for models A and B were 0.816 (95%CI: 0.766–0.861) and 0.805 (95%CI: 0.7248–0.7571), respectively, indicating that Delphi-based variable selection did not materially compromise predictive performance.


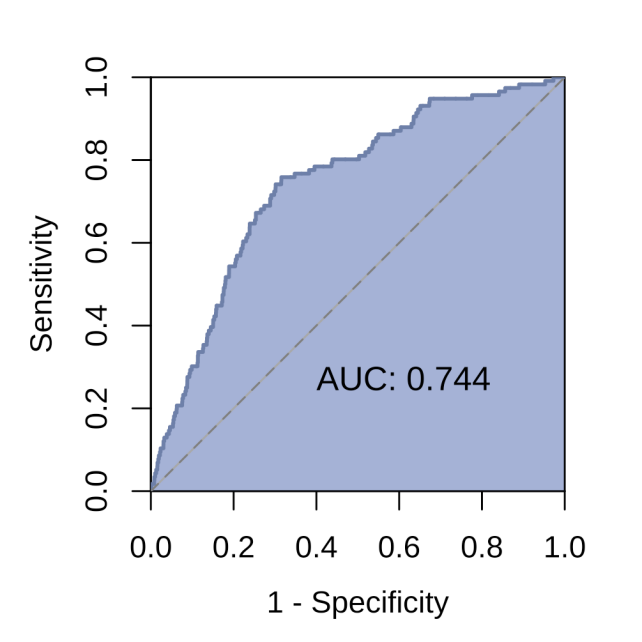


Figure S2. ROC curve of the logistic regression model for predicting GNB‑BSI in the external MIMIC‑IV validation cohort. The external cohort included 4,932 patients, of whom 116 (2.35%) had GNB‑BSI. Because PCT was unavailable in MIMIC‑IV, the logistic regression model was applied using six predictors (age, venous_catheter, LOS, min_wbc, max_crp, and max_neutrophil_rate). The model achieved an AUC of 0.744 (95%CI: 0.700–0.787). The corresponding performance metrics (recall 0.647, accuracy 0.755, specificity 0.757) are provided in Supplementary Table S6.


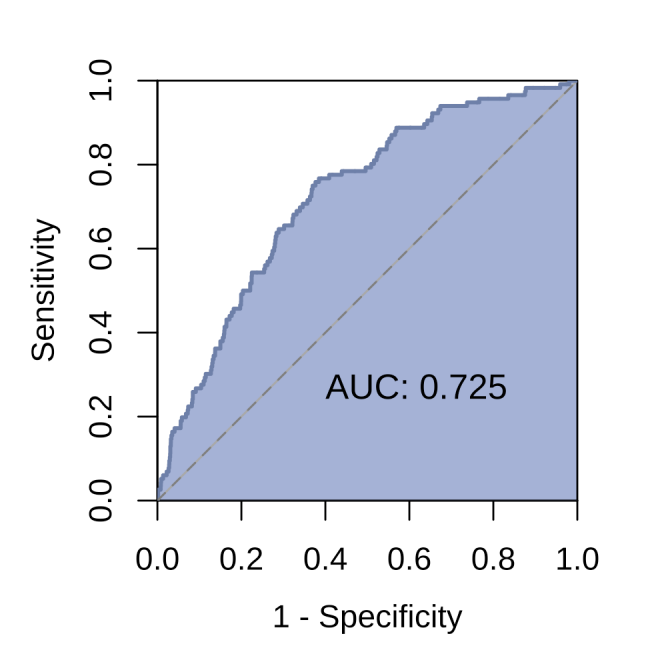


Figure S3. ROC curve of external validation for the sensitivity analysis (complete-case analysis). The logistic regression retrained on the complete-case internal cohort (excluding any patient with missing data in the seven predictors) was validated on the independent MIMIC-IV database. The model yielded an AUC of 0.725 (95% CI: 0.681-0.769), a sensitivity of 0.638, a specificity of 0.715, and an accuracy of 0.713. For comparison, the original model trained on the internally imputed cohort (median imputation) achieved an AUC of 0.744 (95% CI: 0.700–0.787), a recall of 0.647, an accuracy of 0.755, and a specificity of 0.757 on the same external validation set. This demonstrates that the model's predictive performance is consistent across different missing data handling approaches, confirming the robustness of our primary imputation strategy.


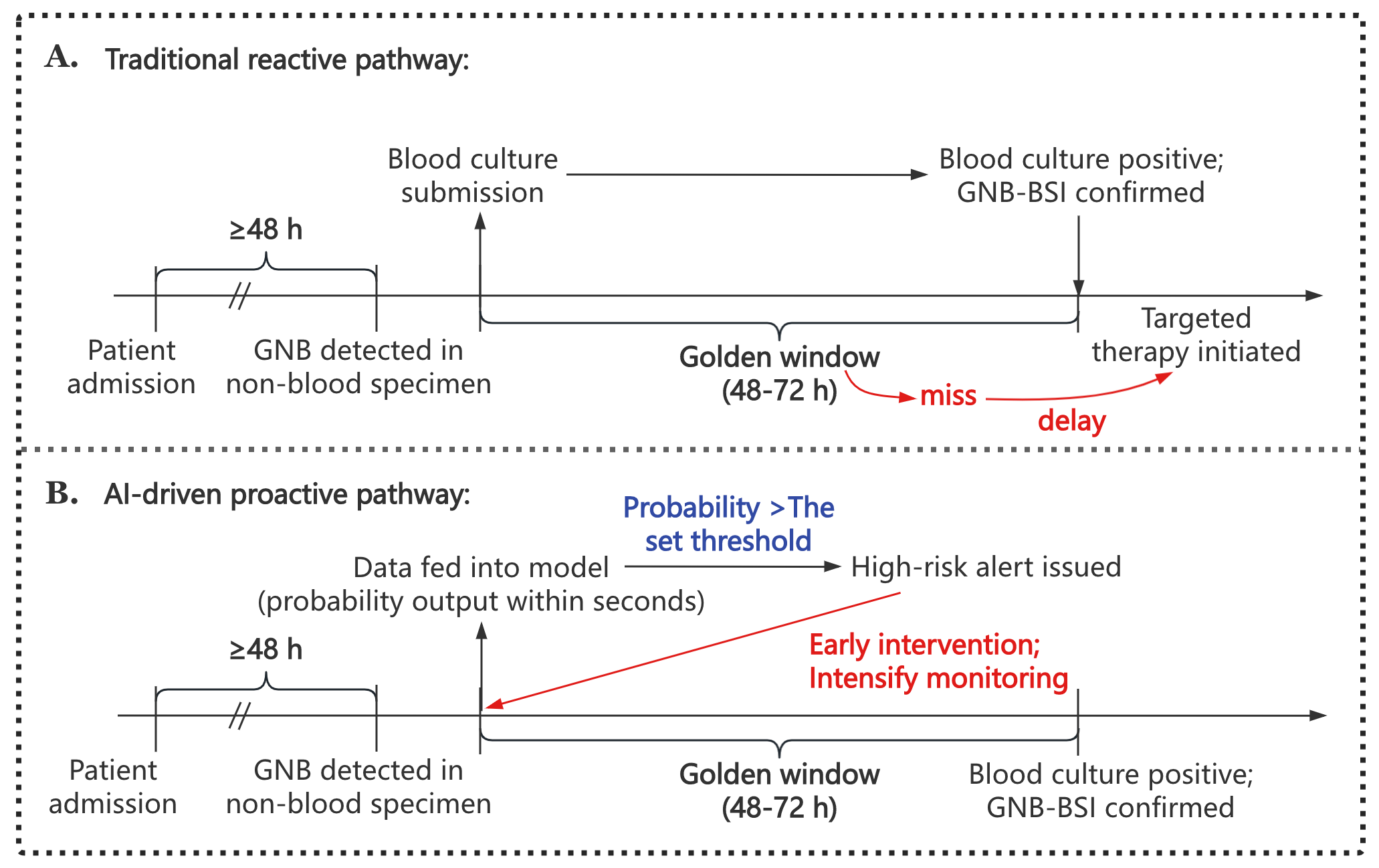


Figure S4. Schematic timeline comparing the traditional reactive pathway (A) and the AI‑driven proactive pathway (B) for the management of GNB‑BSI. By shifting the clinical decision point leftward (48–72 h), the AI‑driven pathway preserves the golden therapeutic window and is expected to improve patient outcomes while reducing healthcare resource utilization.


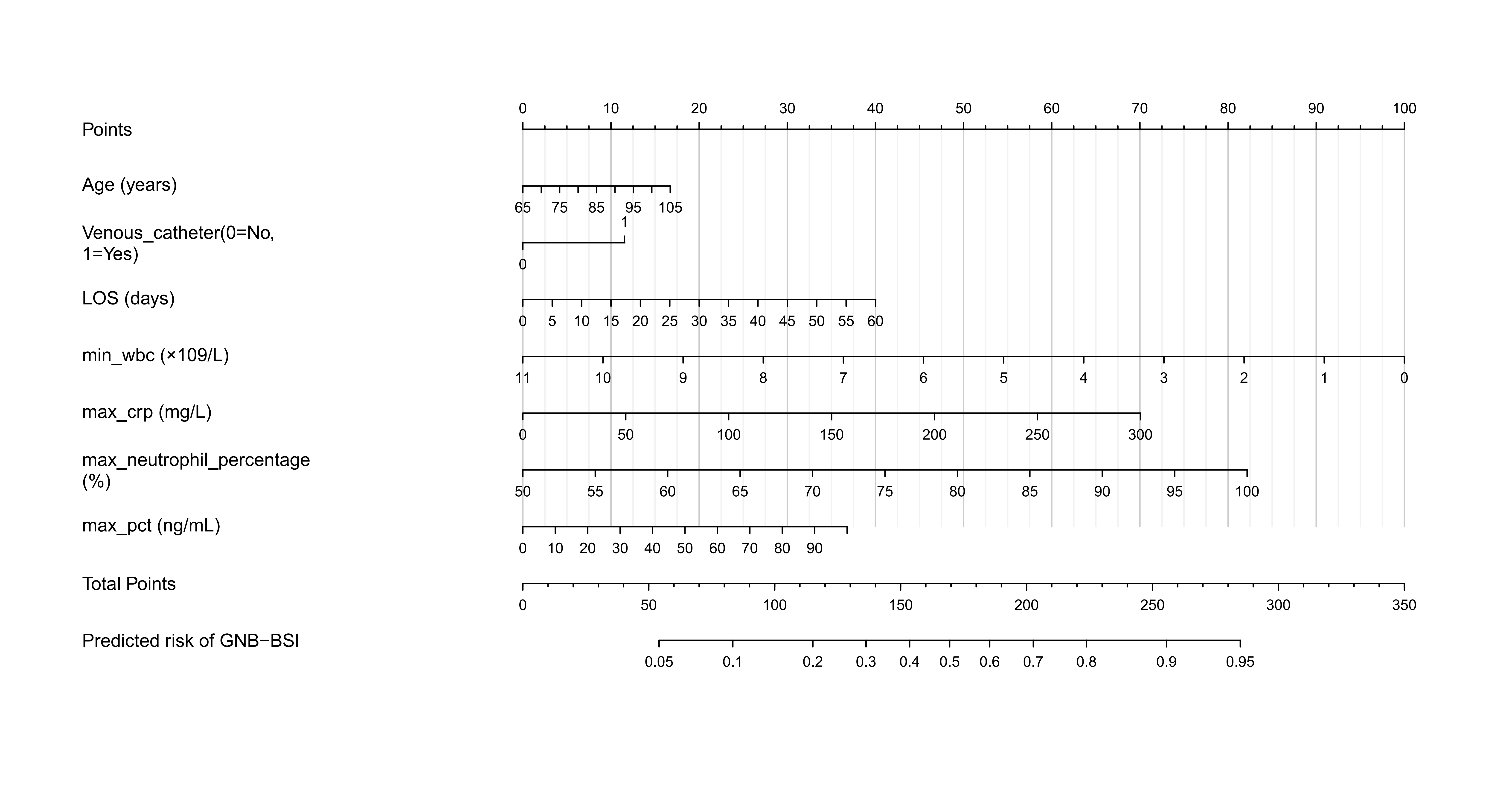


Figure S5. Nomogram for predicting GNB‑BSI in elderly patients after first isolation of GNB-BSI from non‑blood specimens. The nomogram is based on the logistic regression model with the seven predictors listed. To use the nomogram, locate the patient’s value for each predictor on its corresponding axis, draw a vertical line upward to the “Points” scale to obtain the points for that variable. Sum all points to obtain the “Total Points”, then draw a vertical line downward from the total points to the “Predicted risk of GNB‑BSI” scale to read the estimated probability of GNB‑BSI.

The model achieved an AUC of 0.744 (95% CI: 0.700-0.787) in the external test set.
