# Supplementary material for: Predicting Gram-negative bloodstream infection in elderly patients after isolation of GNB from non-blood specimens: a machine learning-based tool
Source: Front Med (Lausanne). 2026 Jun 16;13:1819369. doi: 10.3389/fmed.2026.1819369 (PMC13314445; doi:10.3389/fmed.2026.1819369)
Supplement: Supplementary file 5 [file Supplementary_file_4.docx]

The XGBoost model used booster=gbtree, max_depth=5, eta=0.05, gamma=0.1, subsample=0.8, colsample_bytree=0.8, min_child_weight=1, lambda=2, alpha=1, nrounds=200. The objective function was binary:logistic loss, and AUC was used as the evaluation metric. The random forest model was trained using 500 trees (ntree = 500), a minimum node size of 5 (nodesize = 5), and 10 variables randomly sampled as candidates at each split (mtry = 10). No hard limit was imposed on the maximum number of nodes, allowing trees to grow fully. The importance of each predictor was assessed with 100 permutation replicates (nrep = 100). The artificial neural network was set with size=6, decay=0.01, and maxit=1000. The k-nearest neighbors model used k=7. In decision tree, an initial decision tree was grown on the balanced training set with minsplit=5, minbucket=2, and cp=0. The tree was then pruned using 10-fold cross-validation based on the 1-standard error rule, resulting in a final cp of 0.03125. No explicit depth limit was imposed after pruning. Logistic regression used the default glm settings.
